# PCA-Guided Weakly Supervised Mapping of Hydroxyl- and Iron-Oxide-Related Spectral Anomalies Using Landsat 8 OLI

**DOI:** 10.3390/s26175359

**Published:** 2026-08-25

**Authors:** Kaikai Pang, Yaxiaer Yalikun, Bowen Zhang, Fei Ling, Yilihamujiang Tuniyazi

**Affiliations:** 1 Laboratory of Continental Dynamics and Metallogenic Prognosis of Central Asian Orogenic Belt, Urumqi 830017, China; 2 Urumqi Geological Brigade, Bureau of Geology and Mineral Resources of Xinjiang Uygur Autonomous Region, Urumqi 830017, China

**Keywords:** multispectral sensors, Landsat 8 OLI, weak supervision, spectral–spatial feature extraction, channel attention, hydrothermal alteration mapping

## Abstract

**Highlights:**

**What are the main findings?**

- A PCA-guided weakly supervised framework was developed for Landsat 8 OLI-based mapping of hydroxyl- and iron-oxide-related spectral anomalies.
- The REA-AIE model showed strong agreement with PCA-derived pseudo-labels, with PCA-constrained random sample-level F1 scores of 95.90% and 97.09%, while petrography-constrained site-level assessment detected 43 of 53 altered sites.

**What are the implications of the main findings?**

- The proposed framework provides a practical weakly supervised strategy for multispectral remote sensing data interpretation under limited field-label conditions.
- The refined sensor-derived anomaly maps can support geological investigation by highlighting spectral anomaly zones spatially associated with geological structures for further field verification.

**Abstract:**

Interpreting multispectral remote sensing data for hydrothermal alteration mapping remains challenging in complex mountainous metallogenic belts because dense pixel-level field labels are difficult to obtain and weak spectral responses are affected by lithological background, vegetation, snow/ice cover, and topographic shadow. This study proposes a principal component analysis (PCA)-guided weakly supervised workflow for mapping hydroxyl- and iron-oxide-related spectral anomalies in the Bulong–Maidan–Tuoyun gold–copper metallogenic belt, southwestern Tianshan, China, using Landsat 8 Operational Land Imager (OLI) imagery. PCA was used as a spectral prior to generate PCA-derived positive spectral anomaly samples for model training. A Residual-ECA Alteration Information Extraction (REA-AIE) model was developed to refine PCA-derived anomalies by learning local spectral–spatial features from multispectral image patches. Under the PCA-constrained random sample-level evaluation, REA-AIE achieved F1 scores of 95.90% and 97.09% for hydroxyl- and iron-oxide-related spectral anomalies, respectively; these values indicate agreement with PCA-derived pseudo-labels rather than spatially independent estimates of mapping performance. Petrography-constrained site-level assessment showed that REA-AIE-predicted spectral anomalies occurred within 90 m of 43 of the 53 altered sites, corresponding to a site-level recall of 81.13% and supporting their consistency with field-based geological evidence.

## 1. Introduction

Multispectral satellite sensors provide an important data source for surface material identification and geological information extraction [1]. In particular, Landsat 8 Operational Land Imager (OLI) provides stable multispectral observations across the visible, near-infrared, and shortwave infrared regions, making it suitable for detecting mineral-related spectral responses at a regional scale [2]. Hydroxyl-bearing minerals and iron oxide minerals commonly associated with hydrothermal alteration exhibit diagnostic spectral characteristics within these wavelength ranges [1,3]. Therefore, multispectral sensor data provide useful support for regional mapping of hydroxyl- and iron-oxide-related spectral anomalies in mineral exploration [3]. However, accurate interpretation of alteration-related sensor responses remains challenging in complex mountainous metallogenic belts, where weak spectral signals are easily affected by lithological background, vegetation, snow/ice cover, and topographic shadow [3,4].

Conventional spectral enhancement methods, such as principal component analysis (PCA), band ratio analysis, and minimum noise fraction (MNF) transformation, have been widely used for hydrothermal alteration extraction from multispectral and hyperspectral remote sensing data [4,5,6]. These methods are effective for enhancing mineral-related spectral differences, but their pixel-based nature makes them sensitive to lithological background, illumination variation, and non-target surface materials [1,5,6]. As a result, the extracted anomaly maps often show fragmented or scattered spatial patterns, especially in structurally complex mountainous terrains [5,6].

Deep learning methods, particularly convolutional neural networks (CNNs), provide a way to incorporate local spatial context into remote sensing image interpretation [7]. However, their application to alteration mapping is constrained by the limited availability of dense field labels and by the need to preserve geologically diagnostic spectral information [7,8]. In this context, a practical problem remains unresolved: how to use conventional spectral enhancement results as geologically meaningful weak supervision while refining fragmented spectral anomaly patterns through spectral–spatial feature learning.

Weakly supervised learning has been explored in remote sensing to reduce dependence on dense pixel-level annotations [8]. However, the use of geologically meaningful spectral priors for weakly supervised hydrothermal alteration mapping remains insufficiently investigated. In this study, PCA-derived positive anomaly samples are used as spectral priors for subsequent spectral–spatial refinement [5,8].

To address these bottlenecks, this study develops a PCA-guided weakly supervised framework for spectral anomaly refinement, in which a Residual-ECA network is used to learn local spectral–spatial features from PCA-derived pseudo-label samples. The proposed architecture integrates residual learning with an Efficient Channel Attention (ECA) module. Instead of using channel compression to calculate attention weights, the ECA module directly models local cross-channel interactions in the original channel space through one-dimensional (1D) convolution [9]. This design allows the network to adaptively enhance alteration-related feature channels while preserving channel dimensionality and suppressing background interference from non-mineralized rocks.

**Figure 1:**
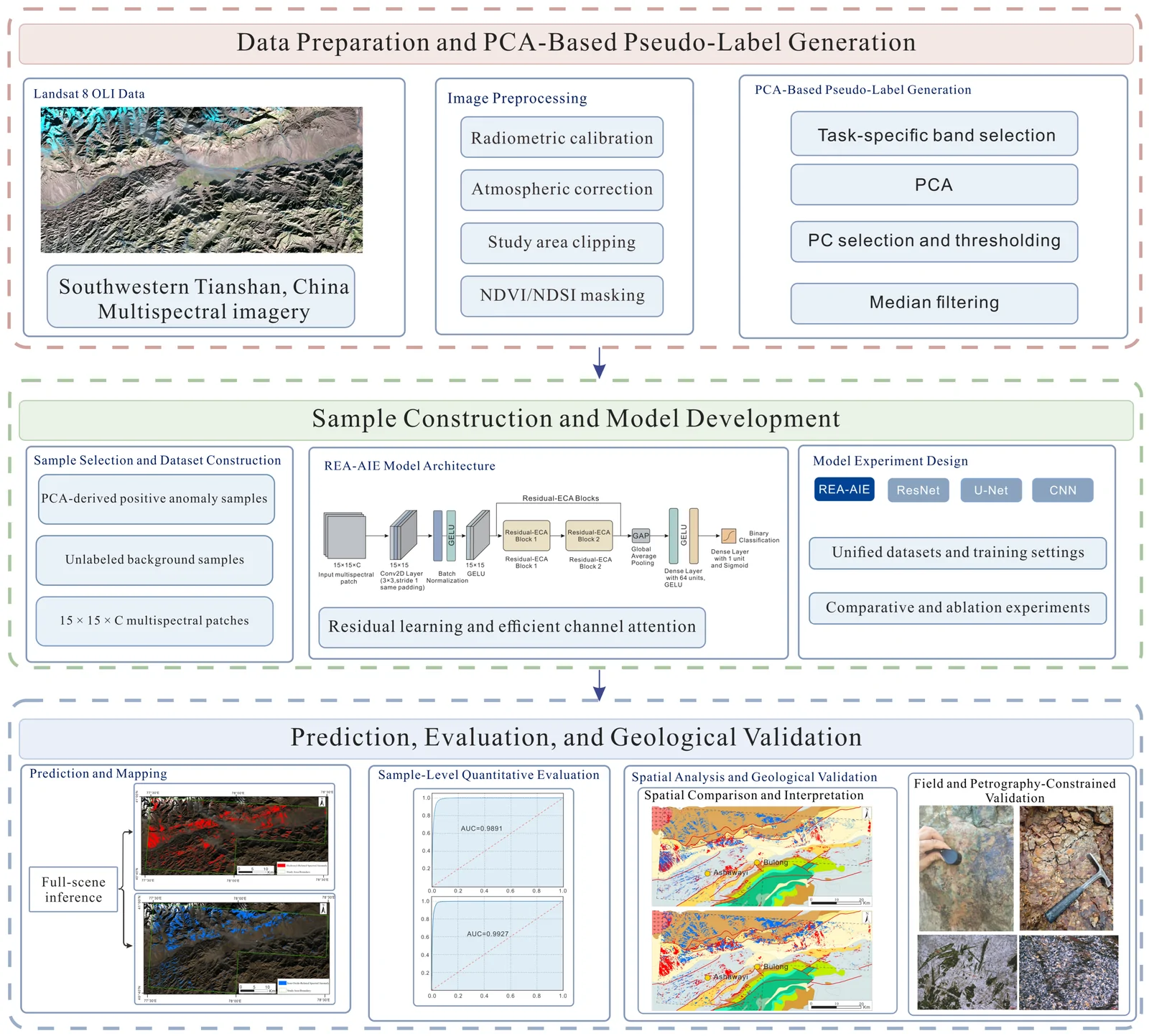
Methodological workflow of this study.

This study makes three main contributions, and the overall workflow is shown in Figure 1. First, a PCA-guided weakly supervised framework is proposed for interpreting Landsat 8 OLI multispectral data under limited field-label conditions. Second, residual learning and an Efficient Channel Attention module are integrated into the proposed network to improve the representation of local spectral–spatial anomaly features. Third, petrography-constrained site-level assessment is introduced to evaluate the geological consistency of the sensor-derived anomaly maps, thereby complementing PCA-constrained random sample-level agreement with pseudo-labels.

## 2. Materials and Methods

### 2.1. Study Area and Geological Setting

**Figure 2:**
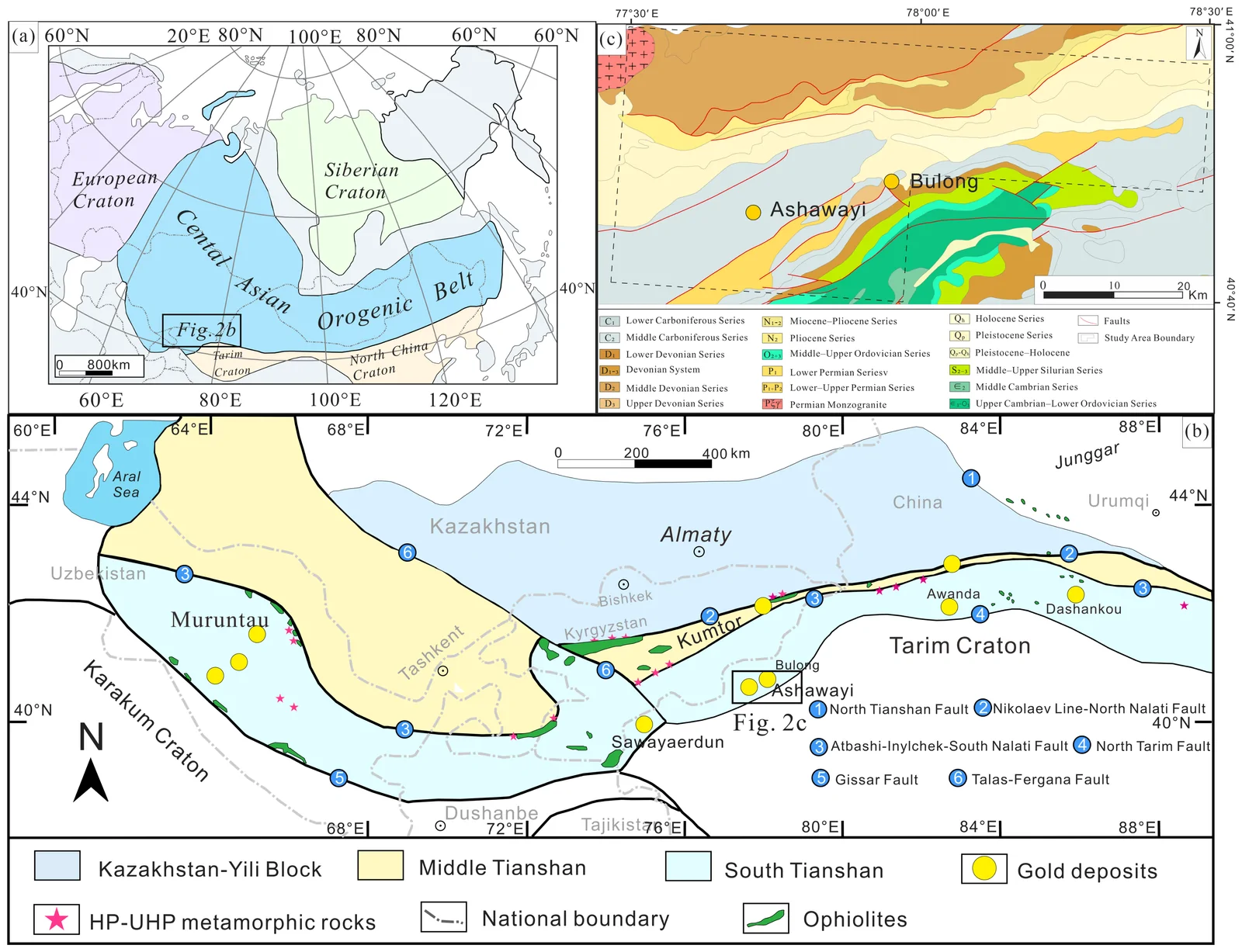
Tectonic location of the study area and its regional geological background. (**a**) Tectonic framework of the Central Asian Orogenic Belt [33]; (**b**) regional position of the study area within the Tianshan tectonic units [34]; (**c**) local distribution of strata, faults, and mineral occurrences in the study area.

The Central Asian Orogenic Belt (CAOB) is one of the world’s largest and most tectonically complex accretionary orogens, recording the prolonged evolution and final closure of the Paleo-Asian Ocean and associated magmatic–hydrothermal activity (Figure 2a) [10,11,12]. Under this regional tectonic framework, the southwestern Tianshan experienced intense tectonic reworking and developed tectono–magmatic–hydrothermal conditions favorable for gold and polymetallic mineralization [13,14,15].

Tectonically, the South Tianshan Orogen records the late Paleozoic subduction and subsequent continental collision between the Tarim Craton and the Kazakhstan–Yili Block, marking the final closure of the Paleo-Asian Ocean (Figure 2b) [16,17,18,19]. This collisional history generated a complex structural framework and extensive magmatic–hydrothermal activity, providing the regional tectonic background for the Bulong–Maidan–Tuoyun gold–copper metallogenic belt [20,21]. Within this belt, Paleozoic clastic and carbonate sedimentary sequences were strongly deformed and locally intruded by magmatic bodies during multiple tectono–thermal episodes [22,23].

These interconnected fault networks and fracture systems acted as primary conduits for the advection of ascending metalliferous hydrothermal fluids, resulting in pervasive wall-rock metasomatism [24,25,26]. Consequently, hydrothermal alteration assemblages, including silicification, hydroxyl-bearing alteration such as sericitization, and supergene oxidation expressed as iron oxide alteration, are commonly developed along structural corridors, fractured zones, and lithological contacts [27,28]. The spatial association between fault systems and alteration halos provides a geological basis for applying remote sensing-based alteration mapping in this region [29,30,31,32].

The Bulong–Maidan–Tuoyun gold–copper metallogenic belt is located in Aheqi County, Kizilsu Kirgiz Autonomous Prefecture, Xinjiang Uygur Autonomous Region. As shown in Figure 2c, strata, faults, mineral occurrences, and mineralization anomalies show a close spatial association, with mineralized features mainly concentrated along fault zones and their adjacent areas, suggesting strong structural control on mineralization. The occurrence of Ashawayi and Bulong near the investigated area indicates favorable regional prospecting potential. Previous geological and mineralogical studies have reported hydrothermal alteration assemblages around these mineralized zones, including silicification, sericitization, chloritization, pyritization, and local carbonate alteration [22,23]. Among these assemblages, sericitization and other hydroxyl-bearing minerals provide a mineralogical basis for hydroxyl-related spectral responses in the shortwave infrared region, whereas weathering and oxidation of Fe-bearing sulfides may produce secondary iron oxides such as limonite and hematite. These alteration and mineralization features provide an important geological basis for interpreting the hydroxyl- and iron-oxide-related spectral anomalies extracted from Landsat 8 OLI data.

Different lithological units occur as belts or irregular blocks in space and have been strongly modified by later tectonic processes. Faults and fracture systems are well developed in the area, providing pathways for hydrothermal fluid migration and favorable sites for the development of wall-rock alteration. Influenced by multistage tectonic–hydrothermal activity, the wall rocks in the study area commonly exhibit varying degrees of alteration, including silicification, iron oxide alteration, and other types of hydrothermal alteration. These alteration features are commonly concentrated along fault zones, fractured belts, and lithological contacts, providing a geological basis for extracting hydroxyl- and iron-oxide-related spectral anomalies from Landsat 8 OLI data. The exposed strata are dominated by Paleozoic sequences, primarily comprising Devonian and Carboniferous formations in the northern part of the study area, with Cambrian–Ordovician and Silurian sequences more widely exposed in the southern part. These stratigraphic units are locally intruded by igneous bodies and have been strongly modified by later tectonic processes.

### 2.2. Data Source and Preprocessing

This study used Landsat 8 Operational Land Imager (OLI) multispectral data as the primary data source for mapping hydroxyl- and iron-oxide-related spectral anomalies in the study area. Compared with other medium-resolution remote sensing datasets, Landsat 8 provides more complete coverage from the visible to near-infrared and shortwave infrared regions, together with stable spatial resolution and high data quality, making it well suited to regional-scale spectral anomaly identification and spatial distribution mapping [2,3,4,35].

**Figure 3:**
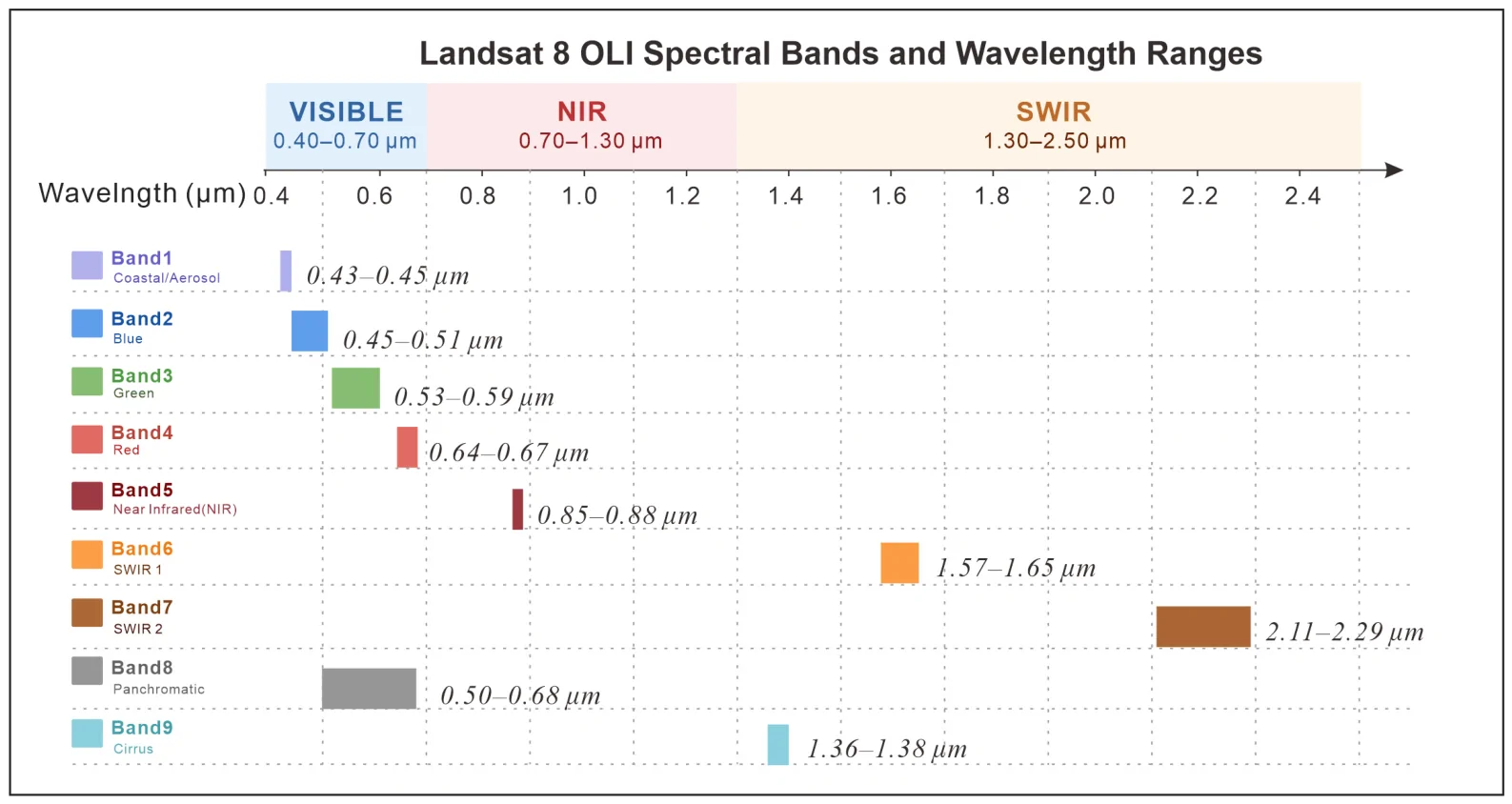
Spectral band configuration of Landsat 8 OLI, showing the wavelength ranges of Bands 1–9 across the visible (VIS), near-infrared (NIR), and shortwave infrared (SWIR) regions.

Landsat 8 OLI provides spectral coverage in the visible, near-infrared, and shortwave infrared regions, making it suitable for alteration-related spectral anomaly extraction [3,36,37]. The main bands and their corresponding wavelength ranges are shown in Figure 3. Bands 1–4 mainly cover the visible region, Band 5 corresponds to the near-infrared region, Bands 6 and 7 fall within the shortwave infrared region, Band 8 is the panchromatic band, and Band 9 is the cirrus band. Because hydroxyl-bearing minerals and iron oxide minerals exhibit distinct spectral responses across these regions, Landsat 8 OLI data provide suitable spectral information for subsequent spectral anomaly extraction [4,38].

The imagery used in this study was obtained from the United States Geological Survey (USGS). The selected Landsat 8 scene was acquired on 11 December 2021, with the product ID LC08_L1TP_148032_20211211_20211216_02_T1. The selected scene has low cloud contamination and generally favorable surface exposure conditions, allowing bedrock spectral information to be represented relatively well, although local topographic shadow and lithological background effects may still influence surface reflectance. The mountainous terrain may introduce residual illumination-related variations in surface reflectance. Topographic correction was not included in the preprocessing workflow. Accordingly, residual slope- and aspect-related illumination effects may remain in the surface-reflectance data and subsequent spectral anomaly products. The selected Level-1TP product provided the geometrically corrected source imagery for subsequent preprocessing and spatial analysis.

To improve the sensitivity of the remote sensing imagery to surface alteration information and, as far as possible, reduce interference from non-target features on spectral characteristics, systematic preprocessing was performed on the Landsat 8 OLI imagery prior to spectral anomaly extraction, including radiometric calibration, atmospheric correction, study-area clipping, and vegetation and snow/ice masking.

The Landsat 8 OLI imagery was first subjected to radiometric calibration to convert the original image values to at-sensor radiance. FLAASH atmospheric correction was subsequently applied to obtain surface reflectance, after which the corrected imagery was clipped to the boundary of the study area. In the FLAASH atmospheric correction, the sensor type was set to Landsat-8 OLI, with a sensor altitude of 705 km and a pixel size of 30 m. The scene center was set to 40°19′44.23″ N and 78°04′43.89″ E, and the acquisition time was set to 05:28:22 GMT on 11 December 2021. The ground elevation was set to 2.058 km. The atmospheric model was set to Sub-Arctic Summer, and the aerosol model was set to Rural. Aerosol retrieval was performed using the 2-Band Over Water option, with an initial visibility of 40 km and a water column multiplier of 1.00. The radiance scale factor was set to 1.000000 for all bands, and the Landsat 8 OLI spectral response function file was used for multispectral channel definition.

The processed image was retained in the WGS 1984/UTM Zone 44N coordinate reference system, with the Transverse Mercator projection, a central meridian of 81.0°, a scale factor of 0.9996, a false easting of 500,000 m, and a false northing of 0 m. Because all reflective bands used in this study were already provided at their native spatial resolution of 30 m and co-registered on the same Level-1TP source grid, no additional resampling was performed during preprocessing or study-area clipping. The final raster grid had a spatial resolution of 30 m × 30 m, no rotation, and an extent from 197,745.0 to 295,065.0 m in the east–west direction and from 4,501,575.0 to 4,553,655.0 m in the north–south direction. After atmospheric correction and study-area clipping, the processed Landsat 8 OLI dataset contained 3244 columns, 1736 rows, and seven reflective bands. All reflective bands used in this study were retained at a spatial resolution of 30 m and aligned on the same raster grid. All subsequent PCA-derived anomaly maps, vegetation and snow/ice masks, pseudo-label maps, prediction-score maps, and site-level assessment layers were generated and maintained on this same raster grid to preserve pixel-level alignment.

**Figure 4:**
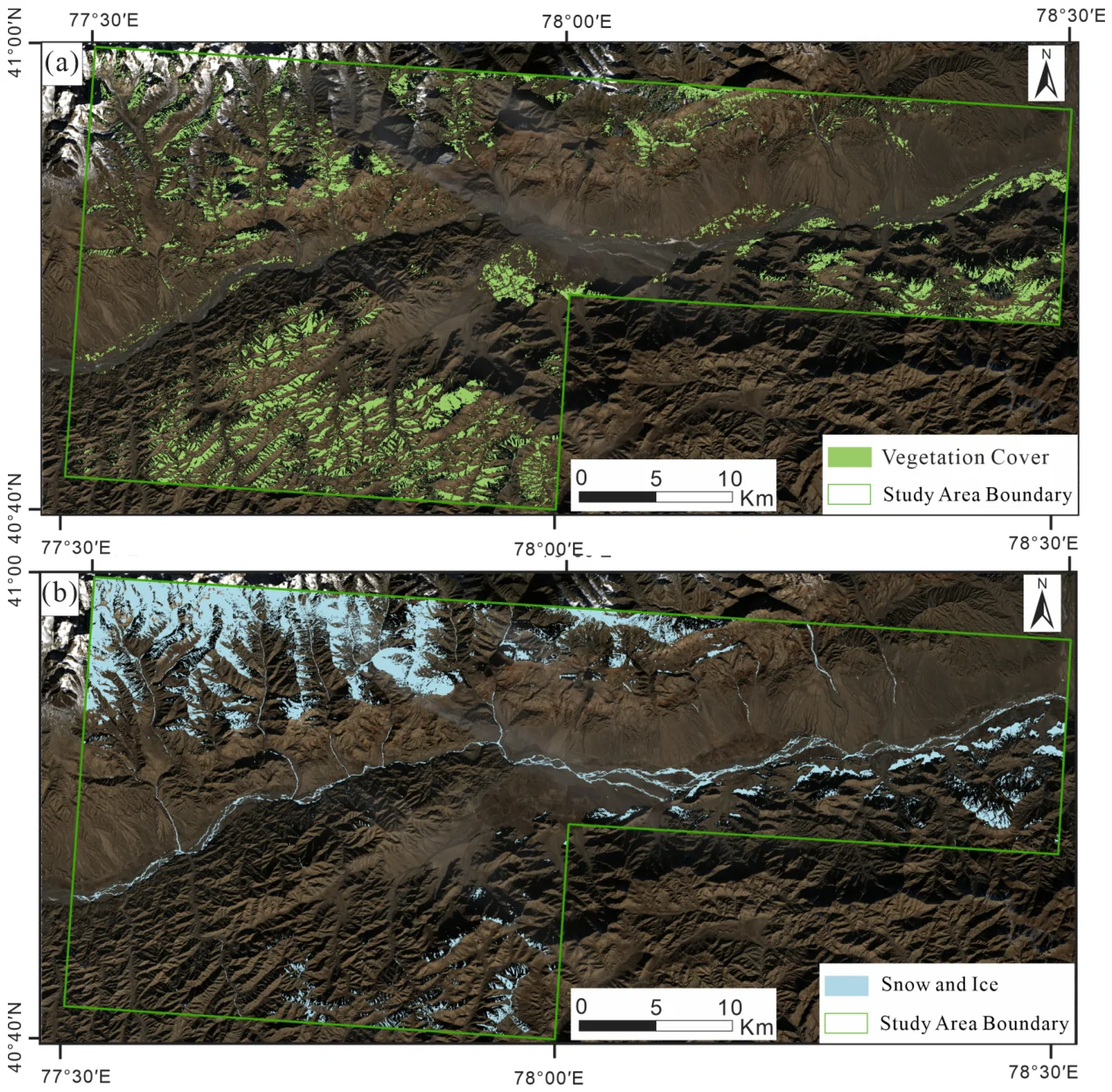
Vegetation and snow/ice masking results for the study area. (**a**) Vegetation mask; (**b**) snow/ice mask.

Vegetation and snow/ice masks were generated using the normalized difference vegetation index (NDVI) and normalized difference snow index (NDSI), respectively. NDVI was calculated as

(1) $$NDVI=\frac{B5-B4}{B5+B4}$$

where B5 and B4 are the near-infrared and red bands of Landsat 8 OLI, respectively. NDSI was calculated as

(2) $$NDSI=\frac{B3-B6}{B3+B6}$$

where B3 and B6 correspond to the green and SWIR1 bands of Landsat 8 OLI, respectively. Based on visual interpretation and repeated threshold testing under the surface-cover conditions of the study area, pixels with NDVI > 0.4 were classified as vegetation-covered areas and masked, whereas pixels with NDSI > 0.4 were classified as snow/ice-covered areas and removed [39,40,41]. To briefly examine threshold sensitivity, nearby thresholds of 0.35 and 0.45 were also visually compared. The main vegetation and snow/ice mask patterns remained broadly consistent, while the threshold of 0.4 provided a balanced removal of non-target cover and retention of exposed bedrock areas. The resulting vegetation and snow/ice masks are shown in Figure 4a,b, respectively. After radiometric calibration, atmospheric correction, and study-area clipping, vegetation and snow/ice masks were applied during PCA-based anomaly extraction. PCA-derived positive anomaly pixels were therefore identified within the unmasked analysis domain. The atmospherically corrected reflectance raster was retained as the multispectral input for subsequent patch construction and model inference. The vegetation and snow/ice masks were used only to reduce surface-cover interference during PCA-based anomaly extraction and did not compensate for terrain-induced illumination differences.

### 2.3. Spectral Anomaly Extraction

Alteration-related spectral anomaly extraction is an important component of remote sensing-based mineral exploration [42]. Its basic principle is to identify alteration-related spectral anomalies by exploiting differences in the spectral responses of alteration minerals in multispectral imagery [43]. The wall rocks in the study area are mainly affected by hydrothermal alteration types such as silicification, sericitization, chloritization, sideritization, and pyritization. Among these alteration types, silicification, pyritization, and sideritization are geologically associated with gold mineralization, providing a mineralization context for interpreting the remotely sensed spectral anomalies. Therefore, the extraction of hydroxyl- and iron-oxide-related spectral anomalies provides useful information for identifying potential zones of hydrothermal activity and mineralization in the study area.

**Figure 5:**
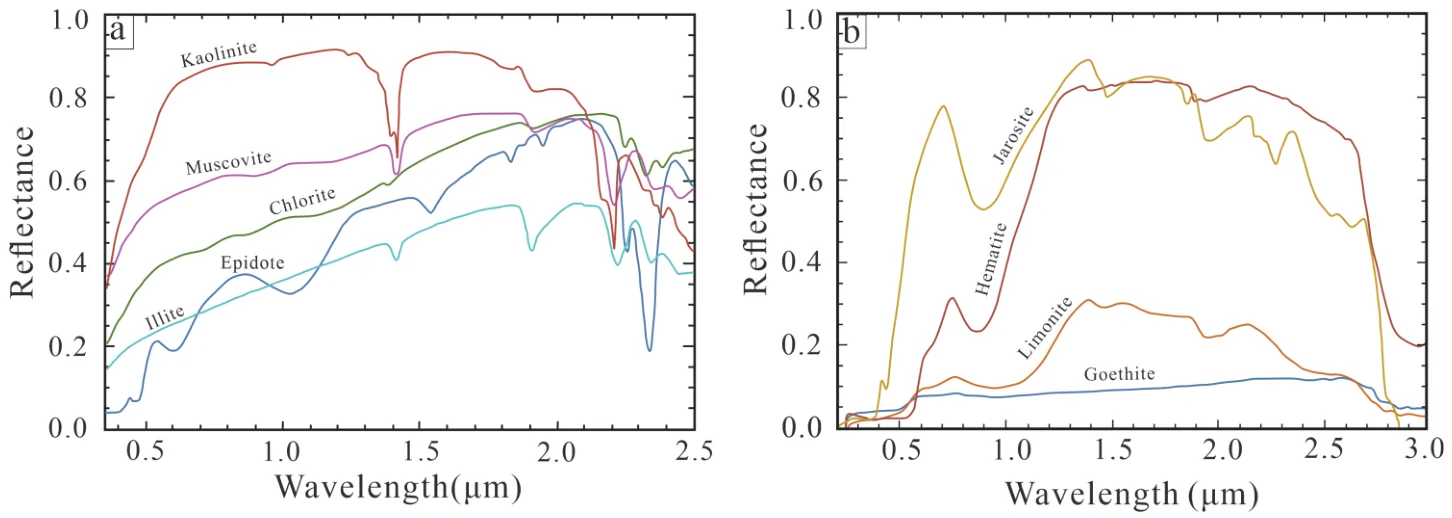
Spectral characteristic curves of typical hydroxyl-bearing minerals and iron oxide minerals. (**a**) Spectral curves of representative hydroxyl-bearing minerals; (**b**) spectral curves of representative iron oxide minerals.

To characterize the spectral response features of different alteration minerals, reference spectral curves of typical hydroxyl-bearing minerals and iron oxide minerals were analyzed in this study, as shown in Figure 5. Figure 5a presents the spectral characteristics of representative hydroxyl-bearing minerals, including kaolinite, muscovite, chlorite, epidote, and illite, whereas Figure 5b shows the spectral characteristics of typical iron oxide minerals, including jarosite, hematite, limonite, and goethite. As can be seen from the figure, hydroxyl-bearing minerals exhibit distinct diagnostic responses in the near-infrared and shortwave infrared regions, particularly near 2.2 μm and the adjacent shortwave infrared wavelengths, where clear spectral differences are observed. In contrast, iron oxide minerals show pronounced reflectance variations in the visible, near-infrared, and parts of the shortwave infrared regions. These spectral differences indicate that hydroxyl- and iron-oxide-related spectral responses can be enhanced and mapped at a regional scale using appropriate combinations of multispectral bands.

Based on the spectral characteristics of the representative alteration minerals shown in Figure 5 and the spectral coverage of Landsat 8 OLI, task-specific band combinations were used for PCA-based spectral anomaly extraction. For the hydroxyl-related spectral anomaly task, OLI Bands 2, 5, 6, and 7 were selected to represent visible, near-infrared, and shortwave-infrared spectral information. For the iron-oxide-related spectral anomaly task, Bands 2, 4, 5, and 6 were selected to emphasize visible-to-shortwave-infrared spectral contrasts relevant to iron oxide responses.

Following the preprocessing described in Section 2.2, PCA was performed separately in ENVI (v5.6, NV5 Geospatial Software, Broomfield, CO, USA) for the two tasks using the valid, unmasked surface-reflectance pixels of the selected bands. The loading characteristics of all principal components were examined to identify components that enhanced the diagnostic spectral contrasts of the corresponding alteration-related responses. The diagnostic loading patterns of the task-specific input bands were used as the primary component-selection criterion, whereas the spatial distribution of extreme component scores in relation to the geological setting was used as a secondary consistency check. The complete PCA loading matrices and explained variance ratios for the two tasks are reported in Table 1 and Table 2.

For the hydroxyl-related spectral anomaly task, PC4 was selected as the anomaly-related component. PC4 accounted for 0.0999% of the total variance and exhibited large, oppositely signed loadings for Bands 6 and 7 (−0.6934 and 0.6587, respectively). This loading pattern emphasized the relatively weak SWIR1–SWIR2 contrast remaining after the dominant common spectral variation had been separated into the preceding components.

For the iron-oxide-related spectral anomaly task, PC4 was also selected as the anomaly-related component. PC4 accounted for 0.1029% of the total variance and showed a strong positive loading for Band 4 (0.7406), together with oppositely signed loadings for Bands 2 and 5 (−0.3921 and −0.5398, respectively). This loading pattern emphasized the visible-to-near-infrared spectral contrast relevant to iron-oxide-related spectral responses.

For both tasks, the original ENVI PC4 orientation was retained, and the positive upper tail was used to represent target-related spectral anomaly responses.

### 2.4. PCA-Constrained Weak Supervision and Dataset Construction

The selected PC4 score maps were used to construct separate pseudo-label datasets for the hydroxyl-related and iron-oxide-related tasks. For each task, the mean and standard deviation of the PC4 scores were calculated using only valid, unmasked pixels. Pixels with scores greater than the mean plus 2.5 standard deviations (μ + 2.5σ) were initially identified as positive anomaly pixels. Vegetation-covered, snow/ice-covered, invalid, and missing pixels were excluded from the calculation of the score statistics and thresholds and were retained as invalid pixels in the resulting maps.

Pixels satisfying the μ + 2.5σ criterion were assigned a value of 1, whereas the remaining valid pixels were assigned a value of 0, thereby generating initial PCA-derived binary anomaly maps. Median filtering was subsequently applied to these initial binary maps to reduce isolated single-pixel responses and generate the final PCA-derived binary anomaly maps. These final binary maps were used both as the source of PCA-derived positive pseudo-labels for model training and as the PCA baseline for subsequent spatial comparison and site-level assessment.

The μ + 2.5σ criterion was adopted as a consistent upper-tail threshold for both tasks to retain relatively high-score component responses and reduce the inclusion of moderate background spectral variability during pseudo-label construction. Following previous studies [6,44], this criterion provides a practical means of identifying candidate spectral anomaly responses where dense field-based labels are unavailable.

The final PCA-derived positive pseudo-label datasets contained 52,180 hydroxyl-related spectral anomaly pixels and 41,328 iron-oxide-related spectral anomaly pixels, giving a total of 93,508 positive anomaly pixels. These values accounted for 0.93% and 0.73% of all pixels in the complete study-area raster for the hydroxyl-related and iron-oxide-related tasks, respectively. The difference in positive-pixel counts represents the mapped extent of the PCA-derived responses under the adopted band combinations, original component orientations, threshold criterion, and filtering procedure; it should not be interpreted as a direct estimate of the relative geological extent of the two alteration types. Separate task-specific datasets were therefore constructed for the subsequent binary classification experiments.

**Figure 6:**
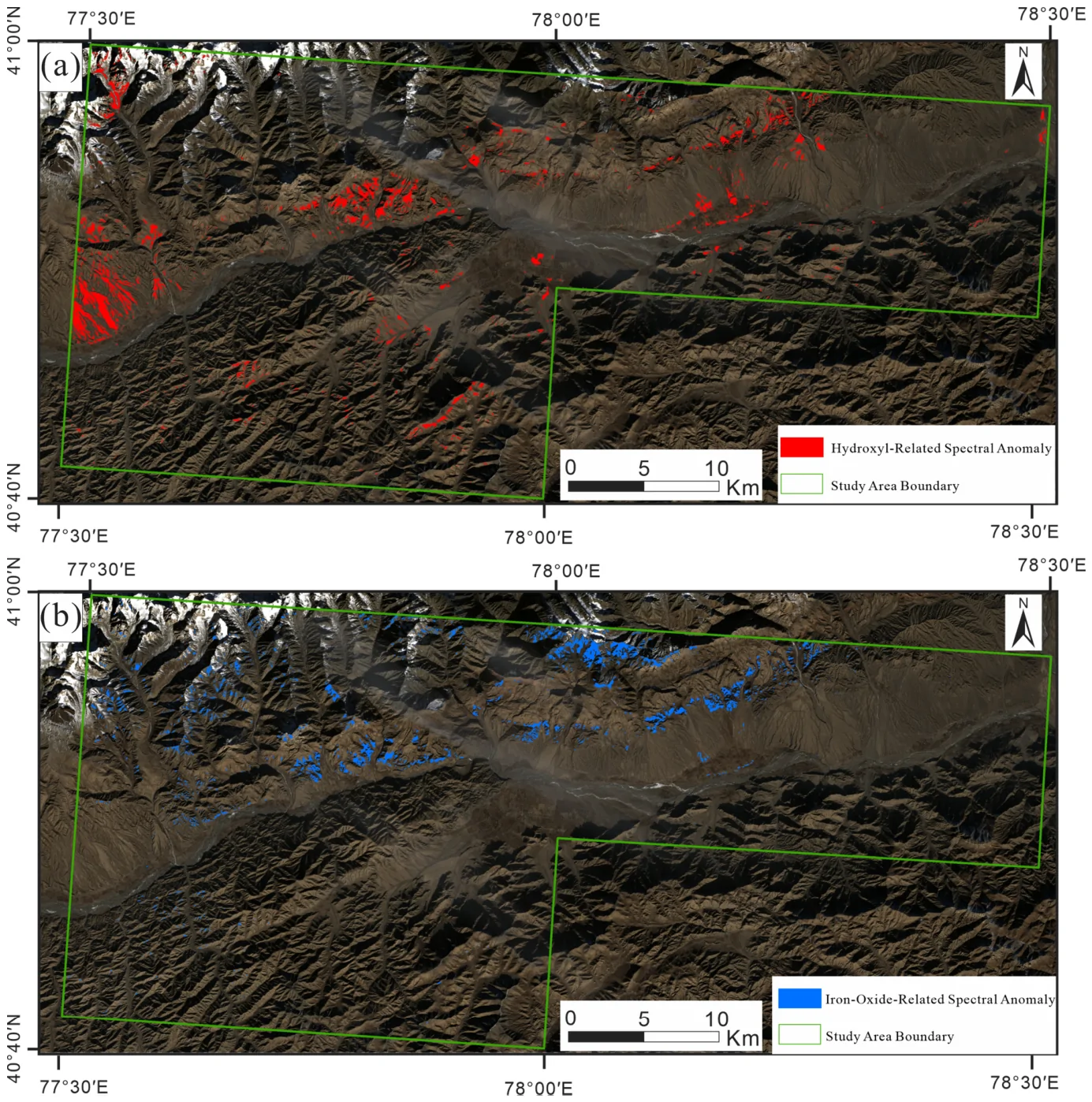
Spatial distribution of PCA-derived positive spectral anomaly samples. (**a**) Hydroxyl-related spectral anomaly samples; (**b**) iron-oxide-related spectral anomaly samples.

As shown in Figure 6, the PCA-derived hydroxyl-related and iron-oxide-related positive spectral anomaly samples display clear differences in spatial distribution. The hydroxyl-related spectral anomaly samples in Figure 6a are mainly distributed in the central–northern and western parts of the study area and generally occur in belt-like, patchy, and irregular areal patterns. In some places, the anomalies form relatively continuous clusters. By contrast, the iron-oxide-related spectral anomaly samples shown in Figure 6b are mainly concentrated in the northern and some central parts of the study area and are characterized predominantly by point-like, patchy, and locally belt-like distributions. Their overall spatial extent is relatively limited and their distribution is more scattered, although local concentration is still observed in some areas. The two types of PCA-derived spectral anomaly samples differ in spatial distribution but also show local adjacency or overlap in certain areas, which may reflect differences in the spectral expression and spatial occurrence of different alteration-related responses in the study area.

In terms of the relationship between the samples and the surface environment, both types of samples are generally concentrated in mountainous areas with well-exposed bedrock and limited vegetation cover, showing good consistency with the topographic conditions and surface exposure characteristics of the study area. This suggests that the vegetation and snow/ice masking applied during preprocessing helped reduce non-target surface interference and that the selected anomaly pixels broadly represent the main spatial distribution patterns of alteration-related spectral information in the study area. In some areas, the sample distribution also shows spatial correspondence with zones of well-developed fault structures, local lithological boundaries, and concentrated anomaly clusters, indicating that the PCA-derived anomaly patterns show spatial correspondence with several geological features and can provide a practical pseudo-label basis for subsequent weakly supervised model training.

For each task, pixels not labeled as PCA-derived positive anomalies formed the candidate unlabeled background pool. These candidate pools contained 5,579,404 pixels for the hydroxyl-related spectral anomaly task and 5,590,256 pixels for the iron-oxide-related spectral anomaly task. From these pools, 52,180 and 41,328 unlabeled background pixels were randomly selected, respectively, to construct balanced task-specific datasets.

These samples were treated as unlabeled background rather than independently confirmed non-altered pixels because locations without PCA-derived anomaly responses may still contain weak alteration-related spectral signals, mixed spectra, poorly exposed alteration, or alteration information not enhanced by the selected principal component. Background candidates were sampled from pixels not labeled as PCA-derived positive anomalies, without an additional spatial buffer around the positive pixels. After balanced sampling, pixels not selected as positive anomaly samples or sampled unlabeled background samples were assigned an internal label of −1 and excluded from model training and sample-level evaluation. For each selected sample, a 15 × 15 multispectral patch centered on the corresponding pixel was extracted from the normalized reflectance raster. The central pixel determined the class label, whereas the surrounding pixels provided local spectral–spatial context. For pixels near the image boundaries, edge-value replication padding was applied to preserve the fixed 15 × 15 patch size. The complete reflectance raster was used for patch construction, including the original reflectance values retained in vegetation- and snow/ice-covered portions of the scene. The normalized raster was first converted into 15 × 15 patches for all pixel centers. The selected positive anomaly and unlabeled background patches were then divided into training and validation subsets using stratified random sampling. Because patches centered on spatially adjacent pixels can overlap, the two subsets may contain shared local spatial information. At the 30 m spatial resolution of Landsat 8 OLI, each 15 × 15 patch covered approximately 450 m × 450 m and provided local spectral–spatial context around the central labeled pixel. Accordingly, the resulting metrics were interpreted as PCA-constrained random sample-level agreement with PCA-derived pseudo-labels rather than spatially independent estimates of geological mapping performance.

### 2.5. REA-AIE Model Architecture

**Figure 7:**
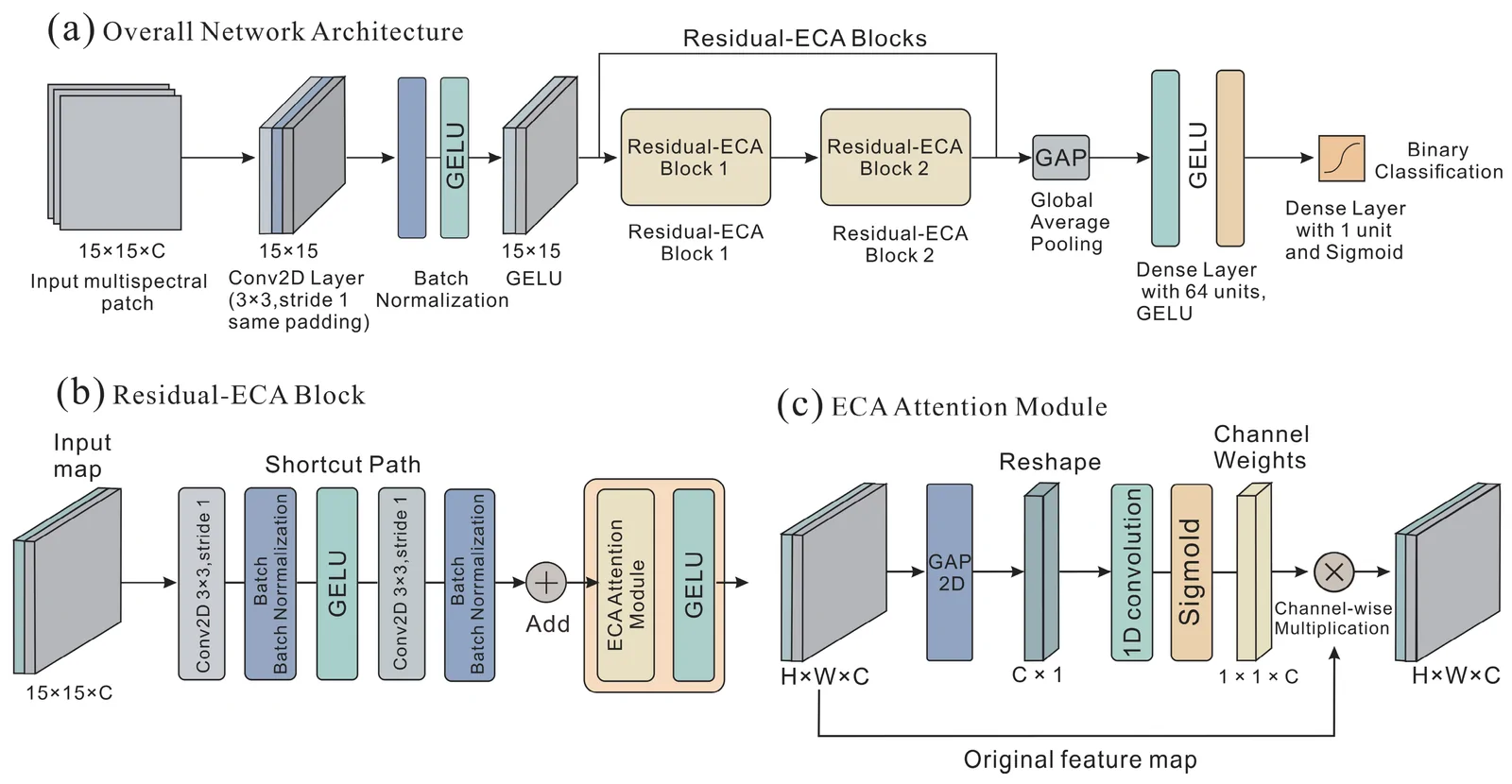
Architecture of the REA-AIE model and its core modules. (**a**) Overall network architecture; (**b**) Residual-ECA Block integrating skip connections with channel attention; (**c**) ECA attention module performing local cross-channel interaction via 1D convolution without channel dimensionality reduction—the key distinction from conventional SE attention. GAP: Global Average Pooling.

To improve the identification of hydroxyl- and iron-oxide-related spectral anomalies under complex mountainous conditions, this study developed the REA-AIE model by integrating residual learning with an Efficient Channel Attention (ECA) mechanism. The model takes normalized multispectral image patches as input and performs spectral anomaly classification through shallow convolutional feature extraction, residual feature propagation, channel-wise attention recalibration, and binary classification. The overall architecture of the REA-AIE model and its core modules are shown in Figure 7. Figure 7a presents the complete network architecture, Figure 7b illustrates the structure of the Residual-ECA Block, and Figure 7c shows the ECA attention module.

In the proposed architecture, each input sample is represented as a 15 × 15 × C multispectral image patch, where C denotes the number of input spectral bands. To keep the network input consistent with the PCA-based spectral anomaly extraction described in Section 2.3, C was set to 4 for both tasks, using OLI Bands 2, 5, 6, and 7 for the hydroxyl-related spectral anomaly task and OLI Bands 2, 4, 5, and 6 for the iron-oxide-related spectral anomaly task. The input patch first passes through a 3 × 3 convolutional layer with 32 filters, stride 1, and same padding, followed by batch normalization and GELU activation. Two Residual-ECA Blocks are then stacked to extract deeper spectral–spatial features while preserving local anomaly boundaries and weak alteration-related information. After the two Residual-ECA Blocks, global average pooling is applied, followed by a fully connected layer with 64 neurons, GELU activation, Dropout with a rate of 0.6, and a final sigmoid classifier for binary spectral anomaly classification.

To describe the computational process of the REA-AIE model more clearly, the main operations of the network can be formulated as follows. Let $X\in R^{H\times W\times C}$ denote the input multispectral image patch, where H and W represent the spatial size of the patch and C denotes the number of input channels. In this study, H=W=15. The initial shallow feature extraction is expressed as

(3) $$F_{0}=\delta \left(\mathrm{BN}\left({\mathrm{Conv}}_{3\times 3}\left(X\right)\right)\right)$$

where ${Conv}_{3\times 3}\left(\cdot \right)$ denotes a 3 × 3 convolution operation, BN(⋅) denotes batch normalization, and δ(⋅) denotes the GELU activation function.

For the l-th Residual-ECA Block, the transformation of the main convolutional branch is given by

(4) $$F\left(F_{l-1}\right)=BN\left(Conv_{3\times 3}\left(\delta \left(BN\left(Conv_{3\times 3}\left(F_{l-1}\right)\right)\right)\right)\right)$$

where $F_{l-1}$ denotes the input feature map of the l-th block. The residual fusion process is then expressed as

(5) $$U_{l}=F_{l-1}+F\left(F_{l-1}\right)$$

where $U_{l}$ is the fused feature map after residual addition. The residual connection helps preserve low-level spatial details and local boundary information while reducing feature degradation during deeper feature extraction.

After residual fusion, channel attention is introduced through the ECA module. First, global average pooling is applied to compress the spatial information of each channel into a channel descriptor:

(6) $$z_{c}=\frac{1}{H_{l}W_{l}}\sum_{i=1}^{H_{l}}\sum_{j=1}^{W_{l}}U_{l}^{c}\left(i,j\right),\;c=1,2,\ldots ,C_{l}$$

where $z_{c}$ is the descriptor of the c-th channel, $U_{l}^{c}$ denotes the c-th channel of $U_{l}$, and $H_{l}$, $W_{l}$, and $C_{l}$ are the height, width, and number of channels of the feature map in the l-th block. The complete channel descriptor is denoted as $z=\left[z_{1},z_{2},\ldots ,z_{C_{l}}\right]$.

In the implemented ECA module, a one-dimensional convolution with kernel size k=3 is used to model local cross-channel interactions without channel dimensionality reduction. The channel attention weights are calculated as

(7) $$w=\sigma \left(Conv1D_{k}\left(z\right)\right),\;k=3$$

where w denotes the channel weight vector, ${Conv1D}_{k}\left(\cdot \right)$ represents one-dimensional convolution with kernel size k, and σ(⋅) denotes the sigmoid activation function.

The recalibrated feature map is obtained by channel-wise multiplication followed by GELU activation:

(8) $$F_{l}=\delta \left(U_{l}⊙w\right)$$

where ⊙ denotes channel-wise multiplication. Through this operation, informative feature channels related to alteration responses are adaptively enhanced, whereas redundant or background-related responses are suppressed.

After two stacked Residual-ECA Blocks, the output feature map is compressed by global average pooling and passed through a fully connected classifier. The final sigmoid output score is expressed as

(9) $$p=\sigma \left(W_{2}Dropout\left(\delta \left(W_{1}GAP\left(F_{2}\right)+b_{1}\right)\right)+b_{2}\right)$$

where $W_{1}$ and $W_{2}$ are the weight matrices of the fully connected layers, $b_{1}$ and $b_{2}$ are the corresponding bias terms, Dropout(⋅) denotes the dropout operation, and p represents the model output score associated with the target anomaly class.

The Residual-ECA Block is the core component of the REA-AIE model. In this block, the residual structure provides a direct shortcut for feature propagation, which helps maintain weak alteration signals and local boundary information during convolutional feature extraction. This is particularly important for spectral anomaly mapping using small image patches because hydroxyl- and iron-oxide-related spectral anomalies in complex mountainous areas often occur as weak, patchy, or belt-like features rather than as isolated single pixels.

The ECA module further improves the channel-wise representation capability of the model. Unlike SE attention, which uses fully connected layers with intermediate channel dimensionality reduction, ECA performs local cross-channel interaction directly in the original channel space through one-dimensional convolution. In the present implementation, the kernel size of the one-dimensional convolution is set to k=3, allowing each feature channel to interact with its neighboring channels while avoiding channel compression. Although ECA operates on learned feature channels rather than directly on the original Landsat bands, these feature channels are derived from multispectral inputs and therefore retain alteration-related spectral–spatial information. This design is suitable for Landsat 8 OLI-based spectral anomaly mapping because hydroxyl-related spectral responses are mainly expressed in the shortwave infrared region, whereas iron-oxide-related spectral responses are more closely associated with visible and near-infrared spectral variations. By preserving channel dimensionality and modeling local channel dependencies, the ECA module enhances weak but diagnostically meaningful alteration-related responses while reducing redundant background information associated with non-mineralized lithologies and other non-target spectral variability. For a fair comparison, the ResNet, U-Net, and CNN baselines were implemented using the same input patches, training samples, loss function, optimizer, and training strategy as the proposed REA-AIE model. The ResNet baseline retained the residual convolutional framework but removed the ECA module to evaluate the contribution of channel attention. The CNN baseline consisted of conventional convolutional layers followed by feature aggregation and fully connected classification layers. Although U-Net was originally designed for pixel-wise semantic segmentation, it was adapted in this study for patch-level binary classification by replacing the original segmentation output layer with a classification head connected to the extracted feature representation.

### 2.6. Experimental Setup and Parameter Configuration

To compare the agreement of the proposed REA-AIE model and benchmark models with PCA-derived pseudo-labels under a unified experimental setting, comparative experiments were conducted using the same data basis, evaluation metrics, and training strategy. The experiments included two PCA-derived pseudo-label tasks, namely hydroxyl-related and iron-oxide-related spectral anomaly identification, and model training, prediction, and sample-level comparison were carried out separately for the two tasks.

The experiments were conducted in an Ubuntu 20.04 environment using Python 3.8, and the models were implemented and trained using TensorFlow (v2.10.0, Google LLC, Mountain View, CA, USA). An NVIDIA RTX A4000 GPU (NVIDIA Corporation, Santa Clara, CA, USA) was used for accelerated computation. The input data consisted of normalized 15 × 15 multispectral image patches, and the task was formulated as a binary classification problem to distinguish PCA-derived positive spectral anomaly samples from selected unlabeled background samples. Before patch extraction, each input band was independently rescaled to the range of 0–1 using the minimum and maximum values of that band in the input raster. The normalized raster was subsequently used for sample construction, model training, validation, and full-area inference. During training, binary cross-entropy was used as the loss function, and the Adam optimizer was adopted with an initial learning rate of $1\times 10^{-4}$. The maximum number of epochs was set to 100, and the batch size was set to 128. To reduce overfitting, Dropout with a rate of 0.6 and L2 regularization were applied. The L2 regularization coefficients were set to $1\times 10^{-4}$ for convolutional layers and $5\times 10^{-4}$ for fully connected layers. Early stopping was implemented by monitoring validation accuracy, with a patience value of 25 epochs, a minimum improvement of 0.0005, and restoration of the weights corresponding to the highest validation accuracy. Model checkpointing followed the same validation-accuracy criterion, and only the model weights corresponding to the highest validation accuracy were retained for subsequent evaluation and inference. In addition, validation loss was monitored using the ReduceLROnPlateau callback. When validation loss did not improve for eight consecutive epochs, the learning rate was reduced by a factor of 0.8, with a minimum learning rate of $1\times 10^{-6}$. Network weights were initialized using the default Glorot uniform initializer in TensorFlow/Keras (v2.10.0). The random seed was fixed at 42 to improve reproducibility. No geometric, spectral, or radiometric data augmentation was applied in the reported experiments. This setting was retained to ensure that all models were compared using the same original PCA-derived samples and input-data distribution. For PCA-constrained sample-level comparison, the sigmoid output was converted into a binary label using a threshold of 0.5 only for calculating threshold-dependent classification metrics on the balanced pseudo-label dataset. This threshold was not used as the final geological mapping criterion. The sigmoid outputs were interpreted as relative prediction scores for ranking anomaly likelihood and for subsequent threshold-based mapping. To examine the sensitivity of mapped spectral anomaly area to threshold selection, prediction-score thresholds from 0.5 to 0.9 were tested. Based on the threshold-sensitivity results and visual inspection of the prediction-score maps, a threshold of 0.7 was adopted as the final mapping threshold. This threshold reduced scattered low-score responses while retaining the principal spatially continuous anomaly zones. The 83 petrographically examined field sites were not used for model training, PCA-derived pseudo-label generation, hyperparameter tuning, or prediction-score threshold selection. The same threshold was applied to both separately trained tasks to provide a consistent mapping criterion for cross-task spatial comparison.

For dataset organization and PCA-constrained sample-level comparison, the balanced labeled samples were divided into training and validation subsets at a ratio of 2:1 using stratified sampling with shuffling enabled and a fixed random seed of 42, ensuring that samples from different classes maintained relatively consistent proportions in both subsets. Considering the differences between the hydroxyl-related and iron-oxide-related spectral anomaly tasks in terms of spectral response characteristics and spatial distribution patterns, two separate experimental tasks were designed, and model training and prediction were conducted independently for each sample type to enhance the model’s task-specific identification of the two spectral anomaly classes.

To assess the performance of the REA-AIE model, ResNet, U-Net, and CNN were selected as benchmark models for comparison. All models were tested using the same dataset, sample partitioning strategy, training setting, and evaluation metrics to ensure comparability. After training, each model was applied across the complete study-area raster using a sliding-window inference strategy. A 15 × 15 patch was generated for every raster pixel with an effective stride of one pixel, and each pixel received a single prediction from the patch centered on that pixel. The sigmoid outputs were reshaped to the original raster dimensions to generate prediction-score maps for the hydroxyl-related and iron-oxide-related spectral anomaly tasks. The final binary anomaly maps were then derived using the adopted mapping threshold. In addition, an ablation experiment was conducted using the same data partition, training strategy, and evaluation metrics to compare the Residual-only, ECA-based, and SE-based variants. All comparison models used identical training settings, including optimizer, learning rate, batch size, dropout strategy, L2 regularization, and early-stopping criteria, to ensure that performance differences mainly reflected architectural differences.

### 2.7. Evaluation Metrics

To evaluate agreement between model predictions and PCA-derived pseudo-labels, Accuracy, Precision, Recall, F1 Score, the Kappa coefficient, receiver operating characteristic (ROC) curves, and the area under the ROC curve (ROC-AUC) were calculated on the balanced random sample-level validation subsets. These metrics were used for controlled comparison among models under the same pseudo-label construction strategy and random sample-level split.

For application-oriented full-area analysis, precision–recall (PR) curves and the area under the PR curve (PR-AUC) were calculated from the continuous REA-AIE prediction-score maps. For the full-area PR analysis, the final median-filtered PCA-derived binary anomaly maps described in Section 2.4 were used as reference labels. These maps were generated by applying the μ + 2.5σ threshold to the selected PC4 score maps, followed by median filtering. All remaining raster pixels outside the PCA-derived positive anomaly masks were used as the reference background class solely for this calculation, rather than being regarded as independently confirmed non-alteration pixels. Therefore, the full-area PR-AUC quantifies ranking agreement with the PCA-derived spectral prior under the class imbalance of the complete study-area raster rather than independent geological mapping accuracy.

For the balanced random sample-level validation, TP denotes the number of PCA-derived positive anomaly samples correctly classified as positive, TN denotes the number of selected unlabeled background samples correctly classified as background, FP denotes the number of selected background samples classified as positive, and FN denotes the number of PCA-derived positive anomaly samples classified as background.

For the task-specific site-level assessments, sampling sites were treated as positive or negative according to the corresponding alteration label. The hydroxyl assessment included 42 positive sites and 41 negative sites, with the negative group consisting of 11 iron-oxide-only sites and 30 non-altered control sites. The iron oxide assessment included 39 positive sites and 44 negative sites, with the negative group consisting of 14 hydroxyl-only sites and 30 non-altered control sites. For the union assessment, the 53 altered sites were treated as positive sites and the 30 non-altered control sites as negative sites.

For each assessment task, TP and FN were defined among the corresponding positive sites, whereas TN and FP were defined among the corresponding task-specific negative sites. A site was classified as positive when the corresponding predicted anomaly occurred within the adopted 90 m buffer. For the union assessment, either a hydroxyl-related spectral anomaly or an iron-oxide-related spectral anomaly within the buffer was regarded as a positive detection. Precision, Recall, Specificity, and Balanced Accuracy were calculated separately for hydroxyl-bearing alteration sites, iron oxide alteration sites, and their union. The 95% confidence intervals of Recall were calculated using the Wilson method to quantify the uncertainty of the site-level detection rates.

To further evaluate the implications of prediction scores for the final mapping application, the proportion and area of study-area raster pixels classified as spectral anomalies were calculated under different prediction-score thresholds. Prediction-score thresholds from 0.5 to 0.9 at an interval of 0.1 were tested for both hydroxyl-related and iron-oxide-related spectral anomaly maps. The mapped spectral anomaly proportion was calculated as the number of raster pixels classified as spectral anomalies divided by the total number of pixels in the study-area raster, and the mapped spectral anomaly area was calculated according to the 30 m spatial resolution of Landsat 8 OLI.

## 3. Results

### 3.1. Sample-Level Agreement with PCA-Derived Pseudo-Labels

To compare the ability of different models to reproduce PCA-derived spectral anomaly patterns in remote sensing-based spectral anomaly identification, ResNet, U-Net, and CNN were selected as benchmark models for comparison, and experiments were conducted separately for the hydroxyl-related and iron-oxide-related spectral anomaly tasks. The sample-level agreement results of different models with PCA-derived pseudo-labels are summarized in Table 3.

In the hydroxyl-related spectral anomaly task, REA-AIE achieved the highest Accuracy, Precision, F1 Score, and Kappa coefficient on the PCA-constrained validation samples, while CNN produced a slightly higher Recall. Nevertheless, REA-AIE showed a better overall balance between target detection, false-positive control, and classification consistency. Compared with ResNet, which showed the second-best overall performance, REA-AIE improved Accuracy, Precision, Recall, F1 Score, and Kappa coefficient by 0.99, 1.58, 0.31, 0.95, and 1.98 percentage points, respectively, indicating better consistency with the PCA-derived pseudo-label distribution under the adopted sample-level comparison. The improvements over U-Net and CNN were even more pronounced, further suggesting that REA-AIE can reproduce PCA-derived spectral anomaly patterns more consistently under the adopted sample-level evaluation framework.

In the iron-oxide-related spectral anomaly task, REA-AIE achieved the highest reported values for Accuracy, Precision, Recall, F1 Score, and Kappa under the adopted PCA-constrained sample-level comparison, reaching 97.05%, 95.74%, 98.48%, 97.09%, and 94.10%, respectively. The Recall of 98.48% indicates that the model reproduced most PCA-derived positive samples, with fewer false negatives relative to the PCA-derived reference labels. Compared with CNN, which showed the second-highest overall results, REA-AIE improved Accuracy, Precision, Recall, F1 Score, and Kappa by 0.88, 1.08, 0.62, 0.86, and 1.76 percentage points, respectively. These results indicate closer overall agreement with the PCA-derived pseudo-label distribution under the adopted benchmark setting. The slightly higher evaluation values obtained for the iron-oxide-related spectral anomaly task than for the hydroxyl-related spectral anomaly task suggest greater apparent separability within the present pseudo-label dataset.

### 3.2. Ablation Study on Residual-ECA Spectral–Spatial Refinement

**Figure 8:**
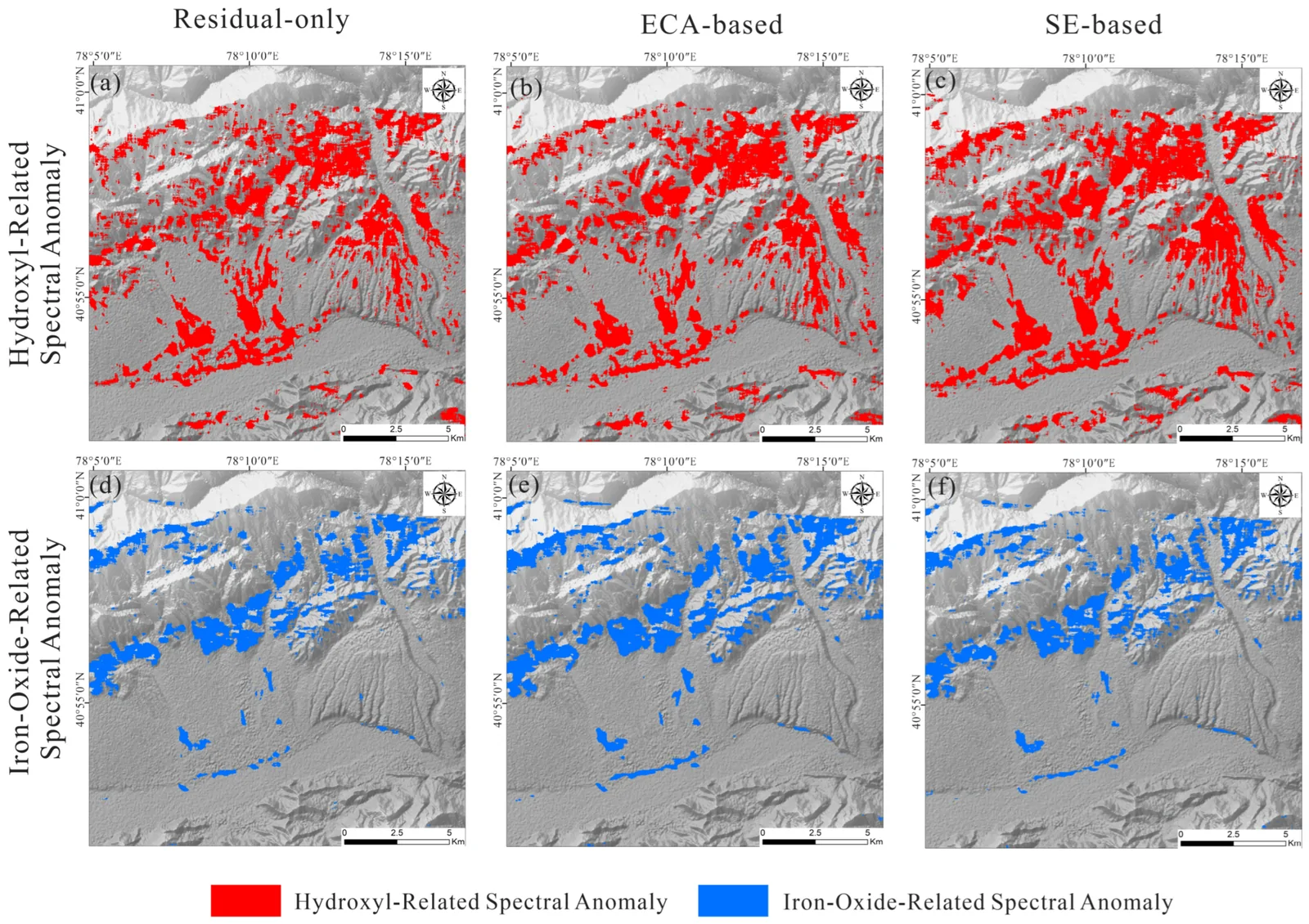
Local spatial comparison of predicted spectral anomaly maps from different attention variants in a representative area. (**a**–**c**) Hydroxyl-related spectral anomaly maps and (**d**–**f**) iron-oxide-related spectral anomaly maps. From left to right: Residual-only, ECA-based, and SE-based variants.

To further evaluate the effect of different model variants on anomaly identification performance, an ablation experiment was conducted under the same data partition, training strategy, and evaluation protocol. Three variants were considered in the ablation experiment: a Residual-only variant without channel attention, an ECA-based variant, and an SE-based variant. For consistency with Table 4 and Figure 8, these variants are denoted as Res-only, Res + ECA, and Res + SE, respectively. For both the hydroxyl-related and iron-oxide-related spectral anomaly tasks, the three variants were trained and evaluated separately using the same dataset, training settings, and evaluation metrics. The quantitative comparison results are summarized in Table 4, and the corresponding local spatial comparison in a representative area is presented in Figure 8. The local comparison area shown in Figure 8 was selected after inspecting the full-area prediction maps, in order to illustrate representative differences in local anomaly continuity and fragmentation among the model variants. This area contains locally developed PCA-derived spectral anomaly responses and continuous prediction patterns and was therefore suitable for visually comparing the spatial refinement effects of the Residual-only, ECA-based, and SE-based variants. It should be noted that this local comparison area was used only for qualitative visual illustration and was not used for model training, hyperparameter tuning, threshold selection, or independent validation.

Table 4 shows that the three variants exhibit different performance characteristics in the two spectral anomaly tasks. In the hydroxyl-related spectral anomaly task, the ECA-based variant achieved the highest Accuracy, Recall, F1 Score, and Kappa coefficient, reaching 95.88%, 96.32%, 95.90%, and 91.76%, respectively, although the Residual-only variant produced a slightly higher Precision value of 95.73%. The improvement in F1 Score from the Residual-only variant to the ECA-based variant was relatively small, indicating that the contribution of ECA was modest for the hydroxyl-related spectral anomaly task. In the iron-oxide-related spectral anomaly task, the ECA-based variant achieved higher Recall, F1 Score, and Kappa coefficient values, whereas the Residual-only variant produced the highest Precision value. These results suggest that ECA mainly changes the balance between target detection and false-positive control rather than uniformly improving all evaluation metrics.

Figure 8 further illustrates the local spatial differences among the three variants. In the hydroxyl-related spectral anomaly maps (Figure 8a–c), the ECA-based variant produces fewer isolated patch-like anomalies and shows better spatial continuity in the main anomaly belts than the Residual-only and SE-based variants. A similar pattern can also be observed in the iron-oxide-related spectral anomaly maps (Figure 8d–f), where the predicted anomalies of the ECA-based variant are more concentrated along structurally favorable zones and the local anomaly patterns appear more organized. Together with the quantitative results in Table 4, these spatial comparisons suggest that ECA may contribute to more coherent local anomaly patterns, although its quantitative contribution to classification performance is modest and task-dependent.

### 3.3. Spatial Characteristics of Predicted Spectral Anomaly Maps Derived from Different Models

**Figure 9:**
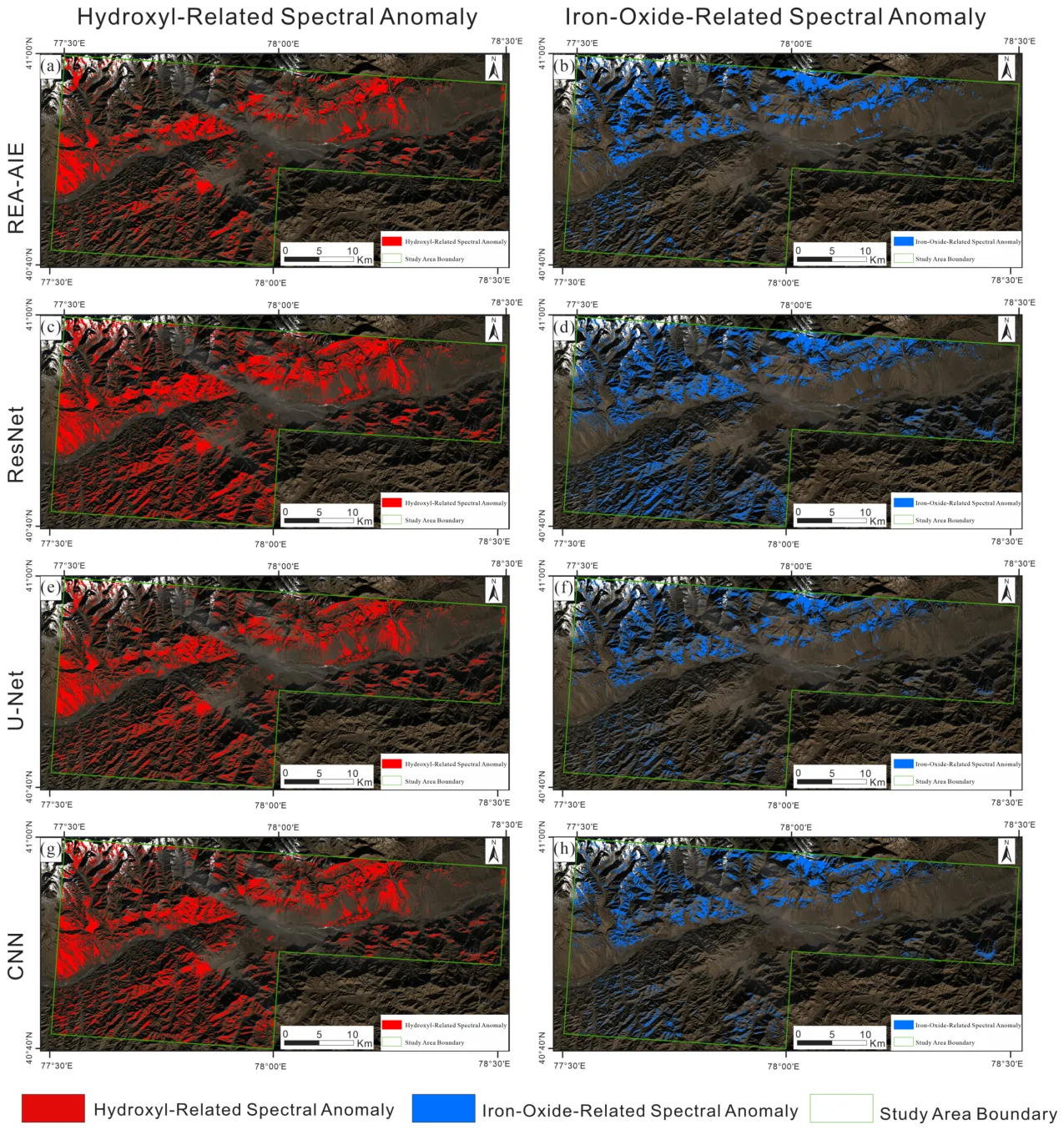
Comparison of the spatial distributions of predicted hydroxyl-related and iron-oxide-related spectral anomaly maps obtained by different models. (**a**,**b**) REA-AIE; (**c**,**d**) ResNet; (**e**,**f**) U-Net; (**g**,**h**) CNN. The left column shows the predicted hydroxyl-related spectral anomaly maps, and the right column shows the predicted iron-oxide-related spectral anomaly maps.

The spatial distributions of the prediction results from different models were compared to assess their ability to identify the two spectral anomaly classes. As shown in Figure 9, Figure 9a,c,e,g present the predicted hydroxyl-related spectral anomaly maps of REA-AIE, ResNet, U-Net, and CNN, respectively, whereas Figure 9b,d,f,h show the corresponding predicted iron-oxide-related spectral anomaly maps of these models. All four models identify the principal spectral anomaly zones within the study area, but they differ markedly in terms of anomaly completeness, boundary delineation, spatial continuity, and background noise suppression.

In the predicted hydroxyl-related spectral anomaly maps, the anomaly zones extracted by REA-AIE are relatively complete, with the main anomaly belts extending continuously across the central–northern and western parts of the study area. These results show good spatial continuity, preserve local details more effectively, and present relatively clear anomaly boundaries. Although ResNet is able to identify most of the major anomaly zones in the study area, it shows anomaly expansion in some areas and relatively coarse local boundaries, suggesting that its ability to constrain background regions is somewhat limited. The main anomaly belts identified by U-Net are generally consistent with those of REA-AIE, but some local areas exhibit scattered noise, and the boundaries of certain anomalies are relatively blurred, resulting in weaker spatial regularity. The main anomaly zones identified by CNN still show a certain degree of continuity, but the overall pattern is more fragmented, with more scattered patches in local areas, indicating that its ability to preserve anomaly integrity and suppress noise under complex background conditions remains limited.

In the predicted iron-oxide-related spectral anomaly maps, spatial differences among the models are also evident. The anomalies extracted by REA-AIE are generally more concentrated, with the main anomaly belts extending continuously across the northern and central–northern parts of the study area. In addition, small-scale local anomalies are better preserved, while scattered anomaly-like responses outside the main structural corridors are relatively limited.

The iron-oxide-related spectral anomaly zones identified by ResNet are relatively overextended, with additional anomaly-like responses appearing in some local areas in the southern and southwestern parts of the study area, resulting in a broader anomaly pattern. Compared with ResNet, the U-Net results are generally more constrained and show reduced background noise, but scattered patches are still present in some local areas, and the continuity of certain anomaly belts remains insufficient. CNN is able to preserve the main locations and overall shapes of the iron-oxide-related spectral anomaly zones to a certain extent; however, compared with REA-AIE, some local anomalies still exhibit a more fragmented pattern, and its background suppression capability is slightly weaker.

The spatial differences among the model outputs are directly associated with their different feature representation capabilities under complex mountainous conditions. In the U-Net and CNN results, the occurrence of scattered patch-like noise indicates insufficient suppression of background interference and weaker preservation of anomaly continuity in spectrally and spatially heterogeneous areas. By contrast, the clearer boundaries and stronger spatial continuity of the REA-AIE results indicate its ability to produce more spatially coherent spectral anomaly patterns. This advantage mainly arises from the coordinated effects of residual connections and the ECA module: the residual structure stabilizes deep feature transmission and helps retain local boundary information, whereas ECA enhances discriminative channels related to alteration responses while reducing interference from irrelevant background features. Consequently, REA-AIE produces anomaly patterns that are more continuous, more spatially coherent, and more consistent with the geological framework of the study area.

As indicated by the spatial distribution patterns in Figure 9, REA-AIE appears to achieve a better balance among anomaly completeness, boundary clarity, and background noise suppression in both spectral anomaly identification tasks. ResNet is more prone to anomaly overexpansion, U-Net still suffers from some local noise interference, and CNN shows a more pronounced fragmented spatial pattern.

### 3.4. Training Stability and Discrimination Performance Under PCA-Constrained Validation

To further compare the classification performance of different models in the hydroxyl-related and iron-oxide-related spectral anomaly tasks, this study analyzes the convergence behavior, class discrimination capability, and misclassification characteristics of REA-AIE, ResNet, U-Net, and CNN by combining the training and validation processes, ROC curves, and confusion matrices. A comprehensive comparison of training stability, discriminative performance, and classification results across the two tasks provides further insight into the differences in applicability of these models for remote sensing-based spectral anomaly identification.

#### 3.4.1. Analysis of the Training Process of Different Models

**Figure 10:**
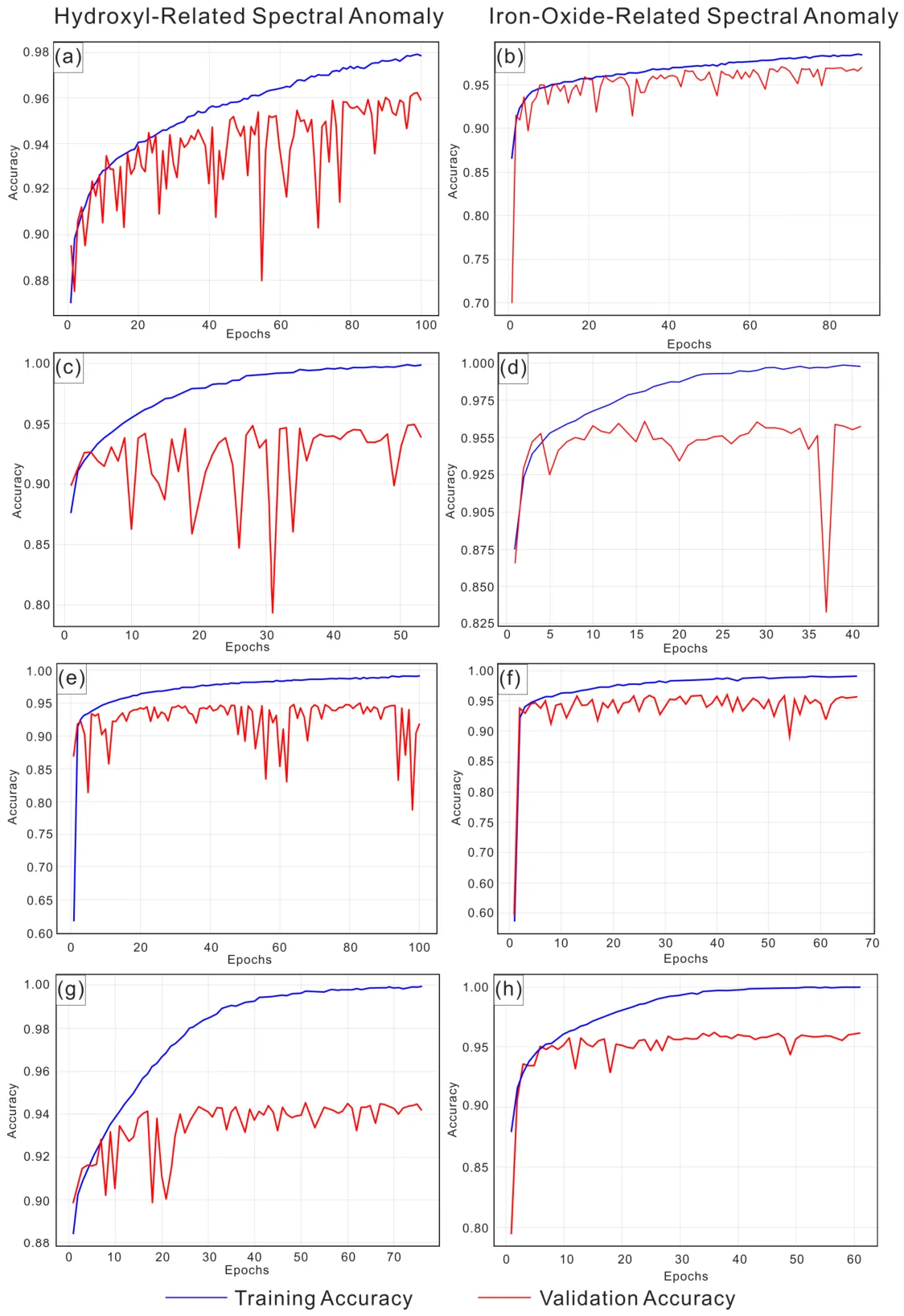
Training and validation accuracy curves of different models for the hydroxyl-related and iron-oxide-related spectral anomaly tasks. (**a**,**b**) REA-AIE; (**c**,**d**) ResNet; (**e**,**f**) U-Net; (**g**,**h**) CNN. The left column corresponds to the hydroxyl-related spectral anomaly task, and the right column corresponds to the iron-oxide-related spectral anomaly task.

The training and validation accuracy curves of the different models for the hydroxyl-related and iron-oxide-related spectral anomaly tasks are shown in Figure 10. Figure 10a,b present the training processes of REA-AIE for the hydroxyl-related and iron-oxide-related spectral anomaly tasks, respectively; Figure 10c,d correspond to ResNet; Figure 10e,f correspond to U-Net; and Figure 10g,h correspond to CNN. Overall, the training accuracy of all four models in both tasks increases continuously with the number of iterations and gradually stabilizes in the later stage, indicating that all models are able to learn the feature characteristics of the training samples. However, clear differences can still be observed among the models in terms of validation accuracy level, fluctuation amplitude, and training stability.

In the hydroxyl-related spectral anomaly task, the training curves of all models generally show a pattern of rapid increase in the early stage followed by gradual convergence, whereas the validation curves exhibit relatively obvious fluctuations. The training accuracy of REA-AIE continues to improve and eventually stabilizes at a high level. Although its validation accuracy shows some fluctuation during training, it remains overall within a relatively high range, and the magnitude of local variation is comparatively well controlled. ResNet shows rapid convergence in training accuracy, but exhibits pronounced dips in the validation curve at multiple epochs, suggesting susceptibility to overfitting on locally complex samples in the hydroxyl-related spectral anomaly task. U-Net is also able to reach a high training accuracy, but its validation curve shows the largest fluctuations, with obvious low values appearing in some stages, suggesting that it is more susceptible to background noise and sample complexity in the hydroxyl-related spectral anomaly task. The training curve of CNN is relatively stable overall, and its accuracy continues to improve in the later stage; however, its overall validation accuracy remains lower than that of REA-AIE, and fluctuations are still present during the early and middle stages, indicating lower validation accuracy and greater validation-curve variability than REA-AIE under the adopted random sample-level split.

In the iron-oxide-related spectral anomaly task, the training and validation processes of all models are generally more stable than those in the hydroxyl-related spectral anomaly task, and the validation accuracy is also consistently higher. For REA-AIE, both training accuracy and validation accuracy increase rapidly within a relatively small number of iterations and remain high and stable in the later stage, demonstrating fast convergence and good training stability. ResNet also shows a rapid increase in training accuracy and gradually approaches saturation, while its validation curve remains at a relatively high level overall, although some noticeable fluctuations can still be observed at certain stages. The validation curve of U-Net in the iron-oxide-related spectral anomaly task is clearly more stable than that in the hydroxyl-related spectral anomaly task, and its overall accuracy is also higher, indicating that this model is better suited to iron-oxide-related spectral anomaly identification than to hydroxyl-related spectral anomaly identification. Both the training and validation curves of CNN are relatively stable and remain at a high level in the later stage, suggesting that it has good training stability in the iron-oxide-related spectral anomaly task, although its overall validation accuracy is still slightly lower than that of REA-AIE.

#### 3.4.2. ROC Curves

**Figure 11:**
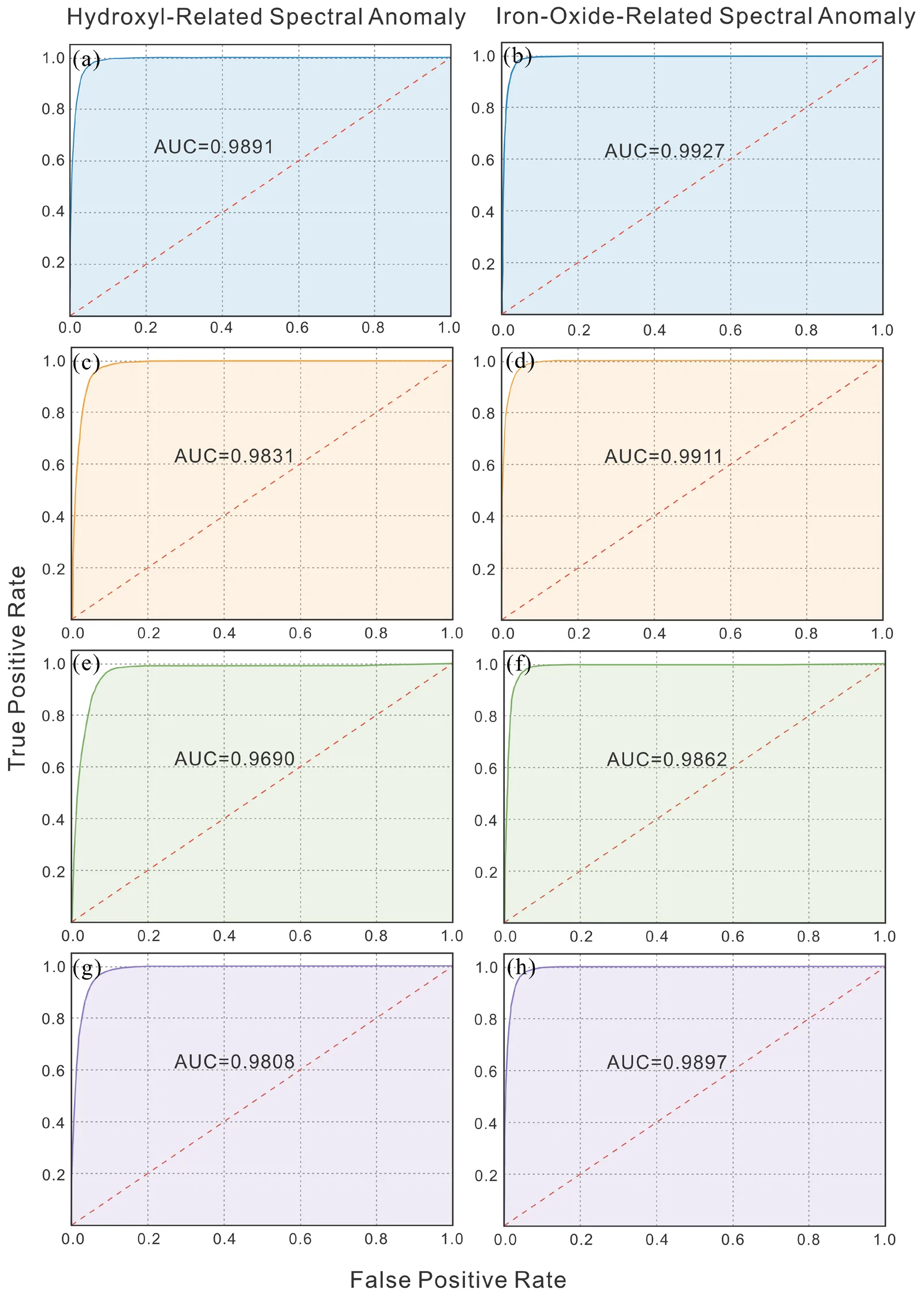
ROC curves of different models for the hydroxyl-related and iron-oxide-related spectral anomaly tasks. (**a**,**b**) REA-AIE; (**c**,**d**) ResNet; (**e**,**f**) U-Net; (**g**,**h**) CNN. The left column corresponds to the hydroxyl-related spectral anomaly task, and the right column corresponds to the iron-oxide-related spectral anomaly task.

The ROC curves of the different models for the hydroxyl-related and iron-oxide-related spectral anomaly tasks are shown in Figure 11. Figure 11a,b present the ROC curves of REA-AIE for the hydroxyl-related and iron-oxide-related spectral anomaly tasks, respectively; Figure 11c,d correspond to ResNet; Figure 11e,f correspond to U-Net; and Figure 11g,h correspond to CNN. Overall, the ROC curves of all four models in both tasks lie above the random classification reference line and are generally close to the upper-left corner, indicating good discrimination capability on the PCA-constrained sample-level evaluation samples. However, differences remain in how closely the curves approach the upper-left corner and in their corresponding AUC values, suggesting that their classification performance is still clearly distinguishable.

In the hydroxyl-related spectral anomaly task, the ROC curve of REA-AIE is closest to the upper-left corner, with an AUC value of 0.9891, indicating the highest class-discrimination performance among the tested models. The ROC curves of ResNet and CNN also show good overall performance, with AUC values of 0.9831 and 0.9808, respectively, suggesting that both models have strong discriminative capability, although their performance remains slightly lower than that of REA-AIE. In contrast, the ROC curve of U-Net is relatively farther from the upper-left corner, with an AUC value of 0.9690, which is the lowest among the four models, indicating that its class discrimination ability is relatively weaker in the hydroxyl-related spectral anomaly task.

In the iron-oxide-related spectral anomaly task, the ROC curves of all models are generally closer to the upper-left corner than those in the hydroxyl-related spectral anomaly task, indicating greater apparent separability between PCA-derived iron-oxide-related positive spectral anomaly samples and unlabeled background samples under the adopted sample-level split. REA-AIE achieved the highest AUC value, reaching 0.9927, which indicates stronger discrimination capability within the PCA-constrained sample-level evaluation framework. The AUC values of ResNet, CNN, and U-Net are 0.9911, 0.9897, and 0.9862, respectively. Although all three benchmark models performed well overall, their validation AUC values were slightly lower than that of REA-AIE. Among them, the ROC curve of ResNet is relatively close in shape to that of REA-AIE, suggesting comparatively strong discriminative performance in the iron-oxide-related spectral anomaly task, whereas U-Net yields the lowest AUC value, indicating that its ability to distinguish iron-oxide-related positive spectral anomaly samples from the background samples remains somewhat weaker than that of the other models.

#### 3.4.3. Confusion Matrix

**Figure 12:**
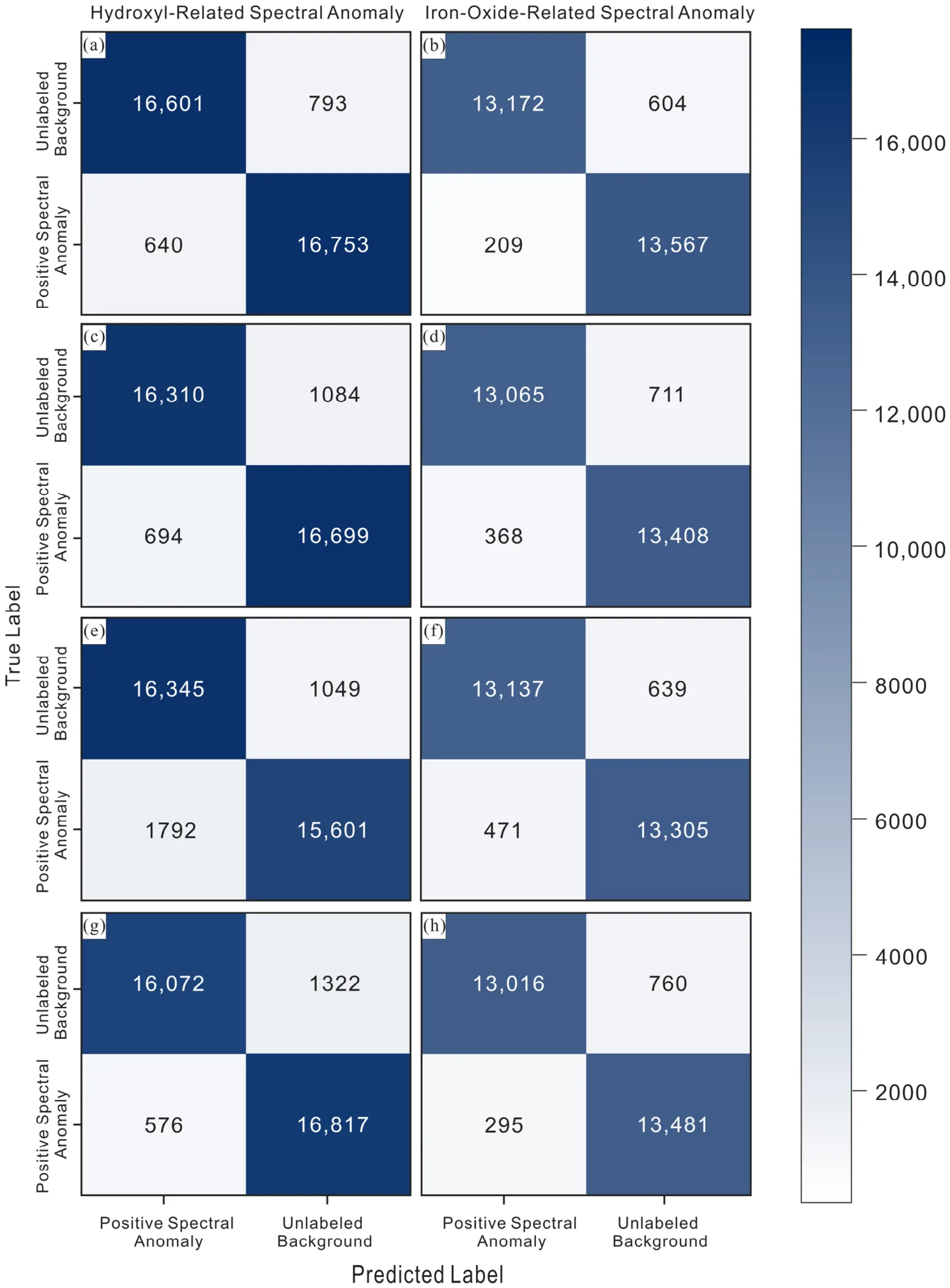
Confusion matrices of different models for the hydroxyl-related and iron-oxide-related spectral anomaly tasks. (**a**,**b**) REA-AIE; (**c**,**d**) ResNet; (**e**,**f**) U-Net; (**g**,**h**) CNN. The left column corresponds to the hydroxyl-related spectral anomaly task, and the right column corresponds to the iron-oxide-related spectral anomaly task.

The confusion matrices of the different models for the hydroxyl-related and iron-oxide-related spectral anomaly tasks are shown in Figure 12. Figure 12a,b present the confusion matrices of REA-AIE for the hydroxyl-related and iron-oxide-related spectral anomaly tasks, respectively; Figure 12c,d correspond to ResNet; Figure 12e,f correspond to U-Net; and Figure 12g,h correspond to CNN. The confusion matrix provides an intuitive view of each model’s correct classifications, false positives, and false negatives for both the unlabeled background class and the positive anomaly class on the validation samples, thereby offering further insight into target detection and background discrimination.

In the hydroxyl-related spectral anomaly task, the confusion matrix of REA-AIE shows that the number of samples on the main diagonal is markedly higher than that on the off-diagonal, indicating strong overall classification performance. Specifically, 16,601 unlabeled background samples and 16,753 positive spectral anomaly samples were correctly classified, whereas 793 unlabeled background samples were misclassified as positive spectral anomalies and 640 positive spectral anomaly samples were misclassified as background. These results indicate that REA-AIE achieves a good balance between background discrimination and target detection under the PCA-constrained validation setting. ResNet also shows relatively good overall classification performance, but the number of misclassified samples is higher than that of REA-AIE, with 1084 false positives and 694 false negatives, suggesting that false detections and missed detections are more evident under complex background conditions. U-Net correctly identifies a relatively large number of background samples, but its number of false negatives reaches 1792, which is significantly higher than that of the other models, indicating a more pronounced omission problem in the hydroxyl-related spectral anomaly task. CNN yields 16,817 true positives, showing relatively strong target detection ability, but it also produces 1322 false positives, the highest among the four models, indicating more false detections in background areas and relatively weaker background suppression capability.

In the iron-oxide-related spectral anomaly task, all models show relatively high numbers of samples along the main diagonal of the confusion matrix, indicating that this task is generally less difficult than the hydroxyl-related spectral anomaly task. REA-AIE produces the fewest misclassified samples, with 604 false positives and 209 false negatives, confirming that it still achieves the fewest overall misclassifications in the iron-oxide-related spectral anomaly task. ResNet performs well overall, with 711 false positives and 368 false negatives, but it still falls short of REA-AIE. U-Net produces relatively few false positives in background samples, with 639 false positives, but its number of false negatives reaches 471, indicating that some iron-oxide-related positive spectral anomaly samples are still classified as background. CNN yields a relatively high number of true positives, with 295 false negatives, suggesting a high true-positive count for iron-oxide-related positive spectral anomaly samples; however, it also produces 760 false positives, the highest among the four models, indicating that its ability to suppress background interference remains weaker than that of REA-AIE and U-Net.

#### 3.4.4. PR-AUC and Threshold-Dependent Mapping Characteristics

Because the PCA-constrained training and validation datasets were artificially balanced, sample-level Accuracy and ROC-AUC may provide an overly favorable view of operational mapping performance under real study-area class imbalance. To provide a more application-oriented evaluation, precision–recall analysis and mapped-area statistics under different prediction-score thresholds were conducted for the final REA-AIE prediction-score maps.

The PR-AUC values were 0.4582 for hydroxyl-related spectral anomaly identification and 0.5457 for iron-oxide-related spectral anomaly identification. Compared with ROC-AUC, PR-AUC is more sensitive to class imbalance and therefore provides complementary information for evaluating full-area ranking agreement with the PCA-derived spectral anomaly reference labels. In addition, the proportion and area of study-area raster pixels classified as spectral anomalies were calculated under prediction-score thresholds from 0.5 to 0.9.

As shown in Table 5, the mapped anomaly area decreased progressively with increasing prediction-score threshold from 0.5 to 0.9. For hydroxyl-related spectral anomalies, the mapped area decreased from 286.7373 km^2^ at a threshold of 0.5 to 171.1692 km^2^ at a threshold of 0.9, corresponding to a decrease in area percentage from 5.6573% to 3.3772%. For iron-oxide-related spectral anomalies, the mapped area decreased from 244.1871 km^2^ to 142.2963 km^2^ over the same threshold range, corresponding to a decrease from 4.8178% to 2.8075%. At the final mapping threshold of 0.7, hydroxyl-related spectral anomalies occupied 238.7871 km^2^, accounting for 4.7113% of the complete study-area raster, whereas iron-oxide-related spectral anomalies occupied 200.5290 km^2^, accounting for 3.9564%. These results provide application-oriented information on the mapped extent of the two spectral anomaly classes under different prediction-score thresholds, complementing the PCA-constrained sample-level accuracy and ROC-AUC results.

### 3.5. Geological Consistency Assessment of Predicted Spectral Anomaly Maps

#### 3.5.1. Field Correspondence of Predicted Spectral Anomalies

**Figure 13:**
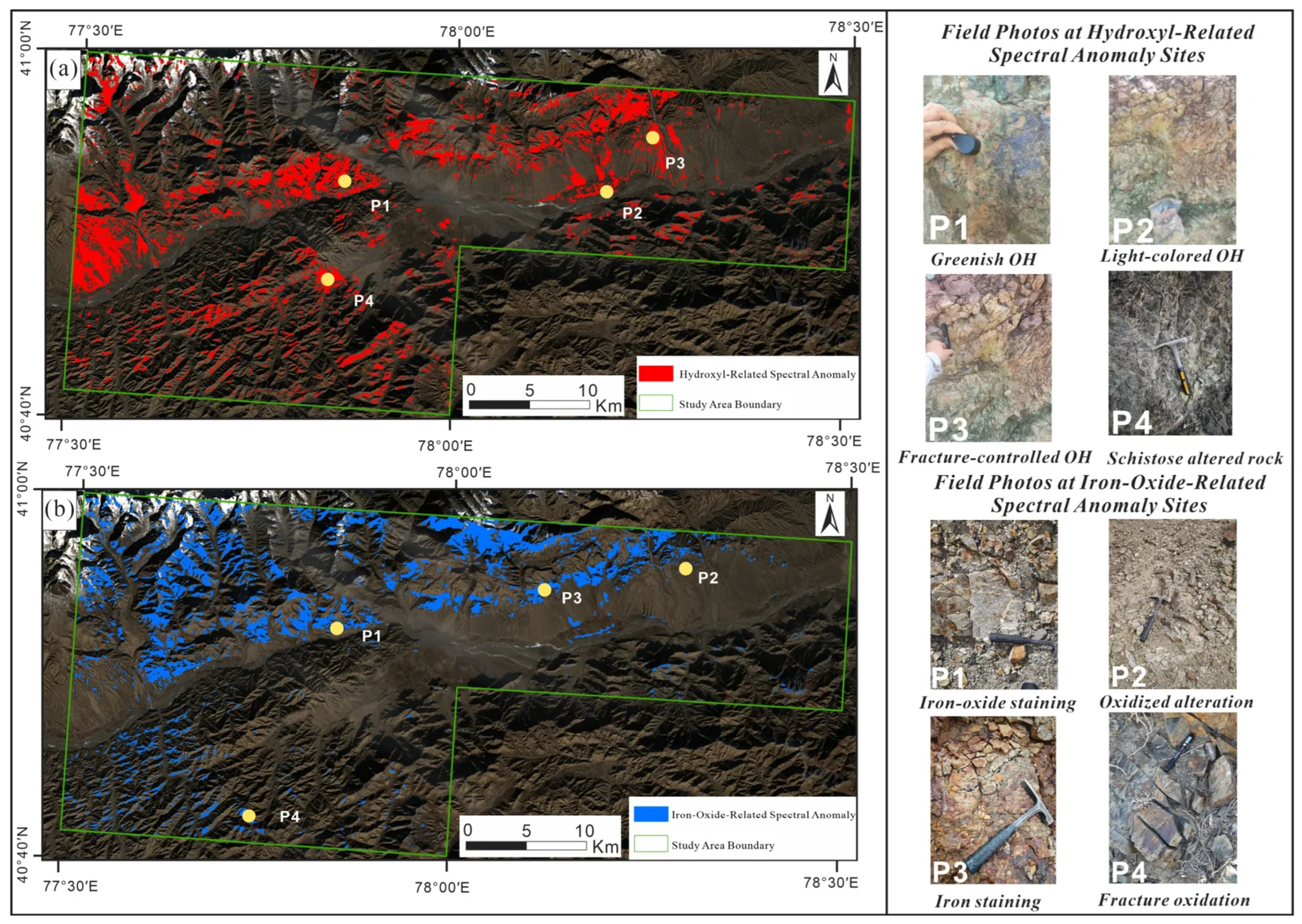
Comparison between the REA-AIE-predicted spectral anomaly maps and field photographs. (**a**) Predicted hydroxyl-related spectral anomaly map and selected field points; (**b**) predicted iron-oxide-related spectral anomaly map and selected field points. The corresponding field photographs are shown on the right.

To qualitatively examine the field correspondence of the predicted anomalies, the predicted hydroxyl-related and iron-oxide-related spectral anomaly maps were compared with field investigation photographs, as shown in Figure 13. Figure 13a presents the predicted hydroxyl-related spectral anomaly map together with the distribution of selected field points, whereas Figure 13b shows the predicted iron-oxide-related spectral anomaly map and the corresponding field points. The field photographs of the selected sites are displayed on the right. The selected verification points are located within or near the anomaly zones predicted by the model and were used to qualitatively assess the consistency between the remote sensing identification results and surface alteration features observed in the field.

In the comparison with the predicted hydroxyl-related spectral anomaly map, P1, P2, P3, and P4 are located within or along the margins of the mapped hydroxyl-related spectral anomaly zones. The field photographs show that P1 exhibits a relatively obvious gray-green alteration, with greenish alteration features developed on the rock surface. P2 is characterized mainly by light-colored alteration, and the rock surface becomes noticeably lighter in color, indicating that the wall rock has undergone relatively strong hydrothermal modification. At P3, the alteration is clearly controlled by fractures and is concentrated along fracture zones and fracture surfaces, suggesting that structural pathways play a direct role in controlling the distribution of alteration. P4 shows foliated altered rocks, with local brecciation and oriented structural features. These field observations are generally consistent with field expressions associated with hydroxyl-bearing alteration, including bleaching, greenish coloration, and structurally controlled alteration. They also correspond to the spatial pattern shown in Figure 13a, where hydroxyl-related spectral anomalies are distributed along structural belts.

In the comparison with the predicted iron-oxide-related spectral anomaly map, P1, P2, P3, and P4 also show good correspondence with the mapped iron-oxide-related spectral anomaly zones. At P1, obvious reddish-brown oxidation staining can be observed, and the rock surface displays prominent ferruginous weathering features. P2 exhibits relatively strong oxidation alteration, with clear color changes on the rock surface. P3 also shows typical iron oxide staining, with reddish-brown staining locally concentrated in certain areas. P4 is characterized by oxidation features developed along fractures, indicating that ferruginous oxidation is more pronounced in structurally fractured zones. These field observations are consistent with the iron-oxide-related spectral anomaly responses in the remote sensing imagery and further suggest that the mapped iron-oxide-related spectral anomalies in the study area are commonly associated with fractures, fractured zones, and locally strongly oxidized areas.

As shown by the verification results in Figure 13, the field manifestations corresponding to the hydroxyl-related spectral anomaly zones are mainly characterized by bleaching, greenish alteration, and structurally controlled altered rocks, whereas the iron-oxide-related spectral anomaly zones are primarily associated with reddish-brown oxidation staining, ferruginous impregnation, and fracture-related oxidation features. Overall, the field observations are consistent with the spatial distribution predicted by the REA-AIE model and provide field-based context for interpreting the identified spectral anomalies.

#### 3.5.2. Petrographic Evidence for Alteration and Mineralization

To further provide mineralogical evidence for interpreting the remote sensing-derived spectral anomalies, microscopic observations were conducted on representative altered and mineralized samples from the study area.

**Figure 14:**
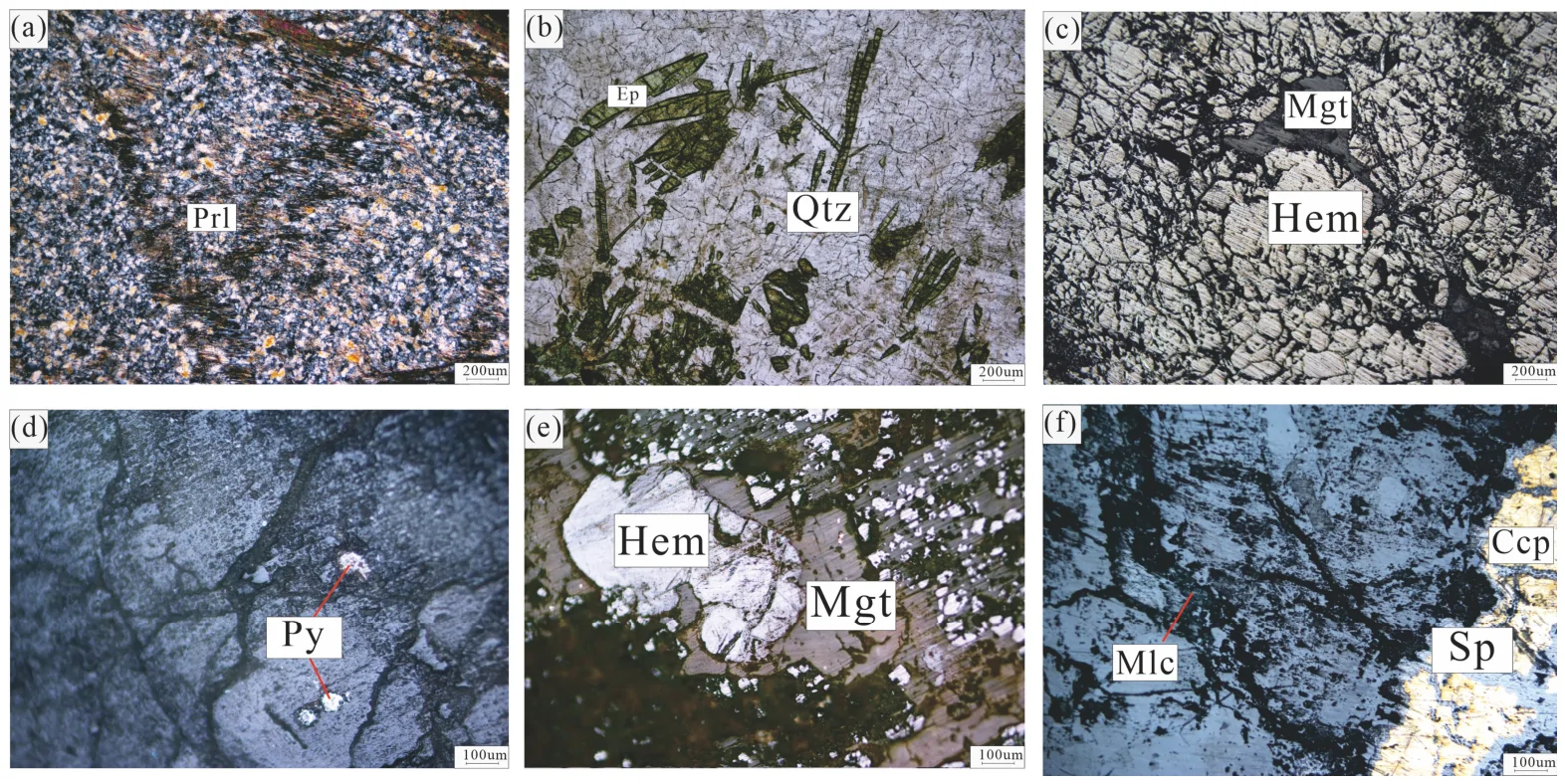
Microscopic evidence of alteration and mineralization in representative samples from the study area. (**a**) Pyrophyllite (Prl), indicating hydroxyl-bearing alteration; (**b**) epidote (Ep) and quartz (Qtz), indicating hydrothermal alteration of wall rocks; (**c**) hematite (Hem) and magnetite (Mgt), supporting iron oxide alteration; (**d**) pyrite (Py), indicating sulfide mineralization; (**e**) hematite (Hem) and magnetite (Mgt), further supporting iron oxide-related mineral assemblages; (**f**) malachite (Mlc), chalcopyrite (Ccp), and sphalerite (Sp), indicating Cu-polymetallic mineralization.

As shown in Figure 14a, pyrophyllite (Prl) was observed in representative altered samples, indicating the presence of hydroxyl-bearing minerals. Epidote (Ep) and quartz (Qtz) in Figure 14b further suggest hydrothermal alteration of wall rocks, which is consistent with the hydroxyl-related spectral anomaly information extracted from the Landsat 8 OLI data. In addition, hematite (Hem) and magnetite (Mgt) are clearly observed in Figure 14c,e, providing direct mineralogical evidence for iron oxide-related alteration. The occurrence of pyrite (Py) in Figure 14d, together with malachite (Mlc), chalcopyrite (Ccp), and sphalerite (Sp) in Figure 14f, indicates that the alteration zones are associated with sulfide mineralization and Cu-polymetallic mineralization. These petrographic observations support the geological consistency of the hydroxyl- and iron-oxide-related spectral anomalies identified by the REA-AIE model and provide additional mineralogical evidence for the exploration significance of the predicted anomaly zones.

#### 3.5.3. Petrography-Constrained Site-Level Assessment

**Figure 15:**
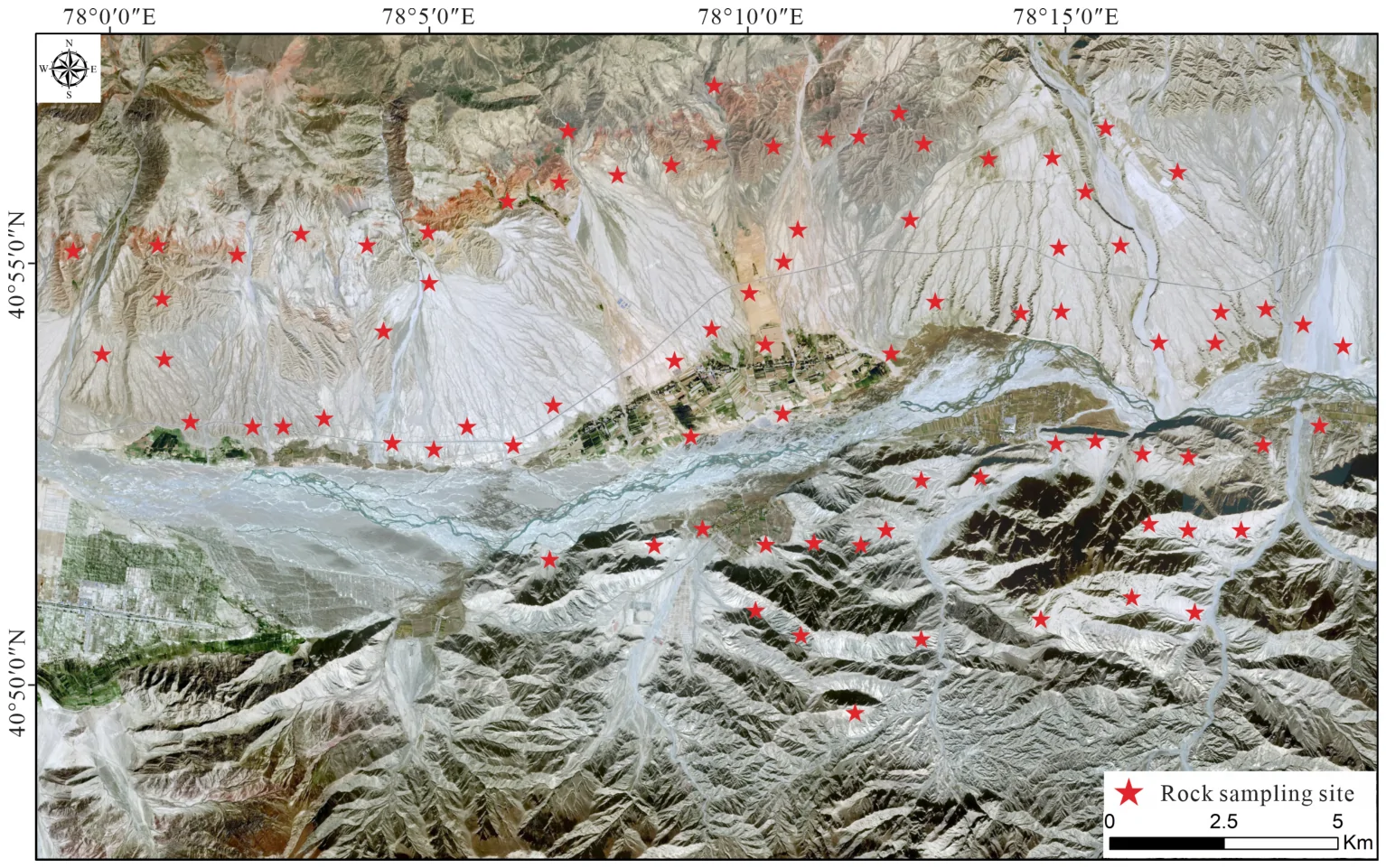
Spatial distribution of rock sampling sites used for petrography-constrained site-level assessment.

To provide an additional assessment of geological consistency, a petrography-constrained site-level assessment was conducted using rock-sampling sites that were independent of model development. The spatial distribution of the sampling sites is shown in Figure 15. The field sampling sites used in this study were collected in August 2025 as part of the geological investigation of the Bulong–Maidan–Tuoyun gold–copper metallogenic belt. The 83 sites used for petrography-constrained site-level assessment represent a selected subset of the available field samples. This subset was selected because these sites had reliable geographic coordinates, complete field descriptions, and petrographic observations sufficient for distinguishing hydroxyl-bearing alteration, iron oxide alteration, and non-altered control samples. Accordingly, this site-level assessment was used to examine geological consistency at the sampled locations rather than to provide complete independent validation of the full-area spectral anomaly maps. The selected sites cover altered rocks, weakly altered rocks, and non-altered control areas across the main lithological units, fault zones, and accessible exposed bedrock areas. Field investigation and petrographic identification were conducted independently of model development. The field and petrographic information was not used for PCA-derived pseudo-label generation, model training, hyperparameter tuning, or prediction-score threshold selection and was used only after the final predicted spectral anomaly maps had been generated for petrography-constrained site-level assessment. A total of 83 rock sampling sites were used for petrography-constrained site-level assessment, including 53 altered sites and 30 non-altered control sites. The site-level reference label of each sampling site was assigned according to field observations and petrographic evidence of alteration- and mineralization-related mineral assemblages. To support the two task-specific spectral anomaly assessments, the 53 altered sites were further classified into hydroxyl-bearing alteration only, iron oxide alteration only, and sites showing both alteration types. Among them, 14 sites were classified as hydroxyl-bearing alteration only, 11 sites as iron oxide alteration only, and 28 sites as showing both hydroxyl-bearing and iron oxide alteration. Accordingly, 42 hydroxyl-bearing alteration sites were used as positive sites for the hydroxyl-related spectral anomaly assessment, and 39 iron oxide alteration sites were used as positive sites for the iron-oxide-related spectral anomaly assessment. Hydroxyl-bearing alteration sites were identified based on field or petrographic evidence of hydroxyl-bearing alteration assemblages, such as sericitization, chloritization, illitization, muscovite- or sericite-bearing alteration, and other hydroxyl-bearing minerals. Iron oxide alteration sites were identified based on visible iron staining, ferruginous oxidation, or petrographic evidence of iron oxide minerals, such as limonite, hematite, goethite, jarosite, or oxidation products related to Fe-bearing sulfides. The 30 non-altered control sites showed no obvious alteration or mineralization-related mineral assemblages in field observations and petrographic inspection.

Considering the 30 m spatial resolution of Landsat 8 OLI and the possible positional uncertainty of field sampling records, a 90 m buffer corresponding to three Landsat 8 OLI pixels was generated around each sampling site. This buffer distance was adopted as a unified site-level assessment criterion to account for the spatial resolution of the imagery, potential positional uncertainty between field sampling locations and image pixels, and the relatively separated spatial distribution of the sampling sites. For the hydroxyl assessment, a hydroxyl-bearing alteration site was regarded as detected when a hydroxyl-related spectral anomaly occurred within the buffer. For the iron oxide assessment, an iron oxide alteration site was regarded as detected when an iron-oxide-related spectral anomaly occurred within the buffer. For the union assessment, an altered site was regarded as detected when either a hydroxyl-related spectral anomaly or an iron-oxide-related spectral anomaly occurred within the buffer. For task-specific negative sites, a false positive was recorded when the corresponding spectral anomaly occurred within the buffer. The median-filtered PCA-derived anomaly maps and the REA-AIE-predicted anomaly maps were compared using the same 90 m buffer criterion.

As shown in Table 6, the petrography-constrained site-level assessment was reported separately for hydroxyl-bearing alteration sites, iron oxide alteration sites, and their union. Under the unified 90 m buffer criterion, REA-AIE detected 34 of the 42 hydroxyl-bearing alteration sites and 32 of the 39 iron oxide alteration sites, corresponding to Recall values of 80.95% and 82.05%, respectively. In comparison, the median-filtered PCA baseline detected 30 of the 42 hydroxyl-bearing alteration sites and 28 of the 39 iron oxide alteration sites, corresponding to Recall values of 71.43% and 71.79%, respectively. In the union assessment, REA-AIE detected 43 of the 53 petrographically verified altered sites, whereas the median-filtered PCA baseline detected 38 altered sites. No false-positive detections were observed among the selected site-level negative samples under the adopted 90 m buffer criterion. These results indicate that REA-AIE improved the recovery of both hydroxyl-bearing and iron oxide alteration sites compared with the median-filtered PCA baseline, while the union assessment provides an overall measure of site-level geological consistency.

Despite the overall site-level correspondence, REA-AIE did not cover 8 of the 42 hydroxyl-bearing alteration sites, 7 of the 39 iron oxide alteration sites, and 10 of the 53 altered sites in the union assessment under the adopted 90 m buffer criterion. These missed sites may be related to weak or mixed spectral responses, limited surface exposure within the corresponding Landsat pixels, residual topographic or lithological interference, or positional mismatch between field records and the 30 m image grid. These possible causes were not independently resolved for each missed site and therefore require further site-specific investigation. In addition, because the 83 sampling sites represent a selected and spatially limited subset of the available field observations, parts of the predicted anomaly maps remain unsampled. These unsampled anomalies should be regarded as unverified remote-sensing anomaly zones requiring future field examination rather than as confirmed alteration occurrences.

## 4. Discussion

### 4.1. Performance Characteristics of REA-AIE and the Role of Channel Attention

**Figure 16:**
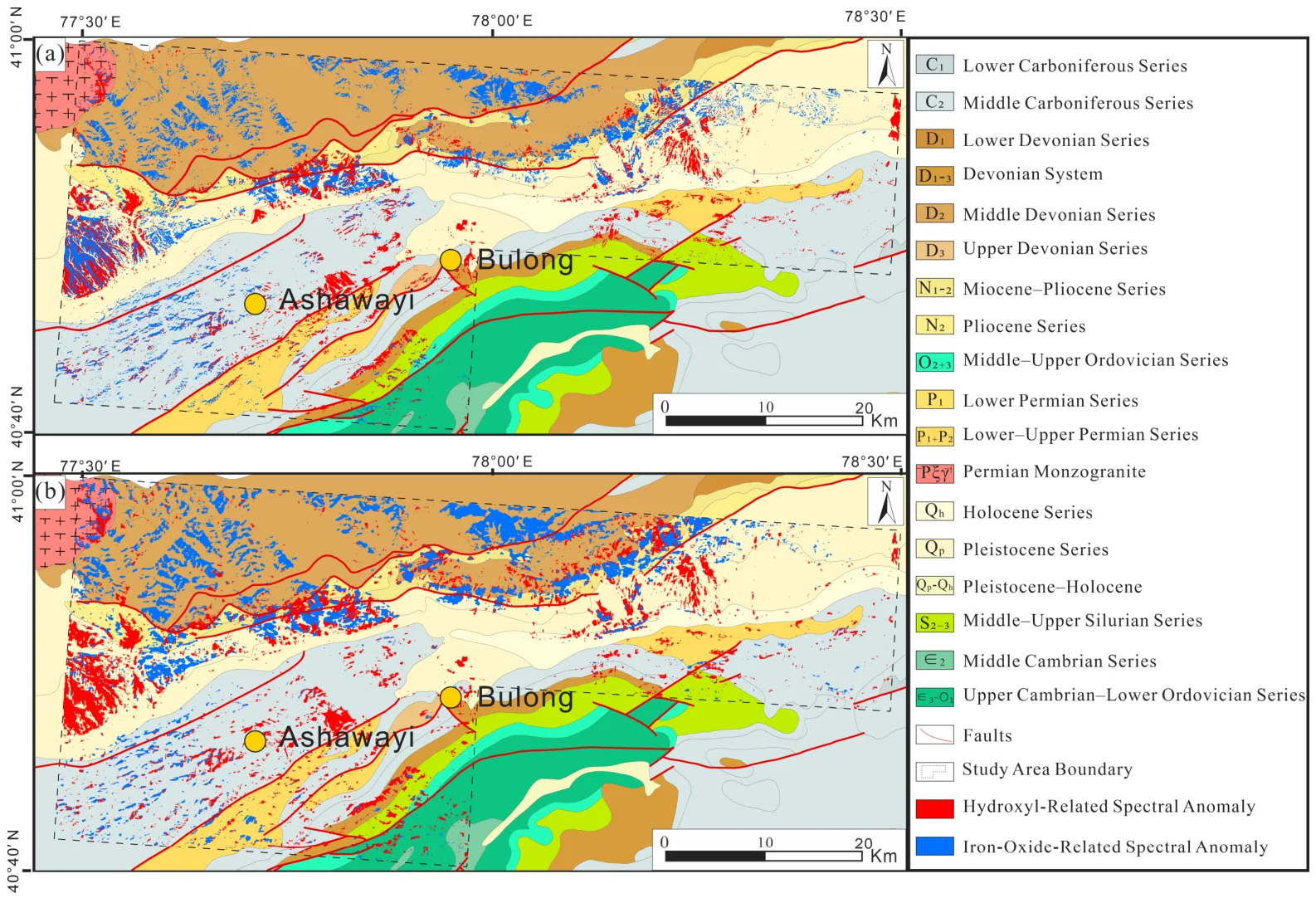
Comparison between median-filtered PCA-derived and REA-AIE-predicted spectral anomaly maps. (**a**) Median-filtered PCA-derived hydroxyl-related and iron-oxide-related spectral anomaly maps; (**b**) REA-AIE-predicted hydroxyl-related and iron-oxide-related spectral anomaly maps.

Figure 16 provides a direct comparison between the median-filtered PCA-derived spectral anomaly maps and the REA-AIE-predicted spectral anomaly maps. The median-filtered PCA result in Figure 16a highlights the major hydroxyl- and iron-oxide-related spectral anomaly responses in the study area, especially along the northern fault zone, the western part of the study area, and the areas surrounding Ashawayi and Bulong. This indicates that PCA is effective for enhancing alteration-related spectral differences in Landsat 8 OLI multispectral data. It should be noted that the PCA-derived binary anomaly maps used for comparison had already been processed using median filtering to reduce isolated single-pixel responses. Therefore, the PCA baseline used in this study represents a simple spatial-refinement baseline rather than a purely unfiltered PCA result. However, the median-filtered PCA-derived anomalies remain relatively scattered in several areas, particularly in the western and northeastern parts of the study area, where fragmented blue and red patches are still distributed. These scattered responses suggest that PCA combined with simple median filtering can enhance alteration-related information and suppress part of the isolated noise but still has limited ability to further constrain anomaly continuity, boundary clarity, and background interference under complex mountainous surface conditions.

This comparison indicates that REA-AIE further reduces isolated patch-like responses relative to the median-filtered PCA-derived anomaly map: the main spectral anomaly belts exhibit greater spatial continuity and are more tightly constrained to structurally favorable corridors.

Compared with the PCA result, the REA-AIE prediction result in Figure 16b preserves the main anomaly centers identified by PCA, while showing better spatial organization. The hydroxyl-related spectral anomalies remain concentrated along the northern and central–northern fault zones, but the distribution is more coherent and less affected by isolated scattered patches. The iron-oxide-related spectral anomalies are also more concentrated around structurally favorable areas, especially near Ashawayi, Bulong, and the fault intersections in the central part of the study area. This indicates that REA-AIE does not simply reproduce or smooth the median-filtered PCA-derived anomaly map; instead, it further refines the initial spectral anomaly information by learning spectral–spatial features from the original multispectral image patches.

The feature-refinement behavior of REA-AIE can be interpreted in relation to its residual learning framework and ECA attention mechanism. The residual connections help maintain stable feature transmission during convolutional feature extraction and reduce feature degradation, which is important for preserving weak alteration signals and local boundary information. More importantly, the ECA module introduces channel attention without channel dimensionality reduction. Unlike some conventional channel attention mechanisms that first compress channel information and then reconstruct channel relationships, ECA models local cross-channel interactions directly in the original channel space. This avoids the potential loss of weak but diagnostically meaningful spectral responses during channel compression. The ablation results further support this interpretation, but their significance lies not only in the numerical differences among variants. The ECA-based variant shows a different balance between target detection and false-positive control, whereas the Residual-only variant achieves slightly higher Precision and therefore exhibits a more conservative prediction behavior. In alteration mapping, especially under complex mountainous conditions, overly conservative predictions may reduce false positives but can also omit weak or spatially transitional spectral anomaly pixels. By contrast, ECA-based channel recalibration reweights alteration-related feature channels through local cross-channel interaction, which may contribute to higher target completeness and more coherent local anomaly patterns. Therefore, the role of ECA should be interpreted primarily as modifying the trade-off between target detection, false-positive control, and local spatial coherence rather than as providing a strong or uniform improvement across all evaluation metrics.

This mechanism may be useful for identifying hydroxyl- and iron-oxide-related spectral anomalies from Landsat 8 OLI data. Hydroxyl-related spectral anomalies are mainly associated with subtle shortwave-infrared responses, whereas iron-oxide-related spectral anomalies are more closely associated with visible-band responses [45]. Both types of spectral responses are susceptible to lithological variation, topographic shadow, sparse vegetation, and exposed non-mineralized surfaces in mountainous regions [46]. By assigning adaptive weights to informative feature channels without reducing channel dimensionality, ECA can reweight alteration-related spectral–spatial feature responses while reducing the influence of redundant background information. Therefore, the clearer anomaly boundaries and reduced fragmented noise in Figure 16b reflect the ability of REA-AIE to transform PCA-constrained spectral anomalies into more spatially coherent and geologically meaningful prediction results.

It should be noted that the sample-level metrics and the petrography-constrained site-level assessment describe different aspects of model performance. The site-level assessment was independent of PCA-derived pseudo-label generation and prediction-score threshold selection and was used only to examine the geological consistency of the final predicted spectral anomaly maps. The F1 scores reported in Table 3 were obtained from the random sample-level split of potentially overlapping image patches and therefore quantify agreement with PCA-derived pseudo-labels rather than spatially independent mapping performance. In contrast, the assessment reported in Table 6 evaluates whether predicted spectral anomalies occur within 90 m of petrographically classified sampling sites. REA-AIE detected 43 of 53 altered sites, corresponding to a site-level recall of 81.13%. Because the sample-level F1 scores and site-level recall were calculated using different reference units and validation designs, they should not be compared numerically. The sample-level metrics are therefore used for controlled cross-model comparison, whereas the petrography-constrained site-level assessment provides an external site-level constraint on geological consistency at the sampled locations. Because the 83 sites constitute a selected and spatially limited subset, this assessment does not represent complete validation of the full-area anomaly maps, and unsampled predicted anomaly zones remain unverified.

In this sense, the performance characteristics of REA-AIE are reflected not only in the sample-level classification metrics but also in the spatial characteristics and geological consistency of the predicted spectral anomalies. Compared with the median-filtered PCA baseline, the REA-AIE results show greater spatial continuity, clearer local boundaries, and reduced fragmented background responses in the mapped anomalies. The comparison in Figure 16, together with the petrography-constrained site-level assessment, therefore suggests that REA-AIE can provide additional spectral–spatial refinement beyond straightforward median filtering in complex mountainous metallogenic belts.

### 4.2. Geodynamic Implications and Structural Controls on Mineralization

The spatial distribution of the spectral anomalies shown in Figure 16 is closely related to the tectonic evolution and fault-controlled mineralization framework of the southwestern Tianshan. The study area lies within the Bulong–Maidan–Tuoyun gold–copper metallogenic belt in the southwestern segment of the South Tianshan. This region experienced long-term oceanic subduction, accretion, collision, and later tectonic reworking associated with the evolution and closure of the Paleo-Asian Ocean. These processes formed a complex fault system and provided favorable structural conditions for hydrothermal fluid migration, wall-rock alteration, and mineralization.

In Figure 16, both hydroxyl- and iron-oxide-related spectral anomalies are mainly concentrated in the northern and central–northern parts of the study area, rather than being randomly distributed across all lithological units. This spatial pattern corresponds closely to the major near east–west-trending faults and their subsidiary structures. In the northern part of the map, spectral anomalies occur along both sides of the main fault zone and are locally strengthened near fault bends and intersections. The repeated spatial association of the blue and red anomalies with the northern fault system is consistent with a possible structural influence on hydrothermal fluid migration and wall-rock alteration. However, this correspondence does not independently demonstrate that the faults acted as the principal fluid pathways.

The anomaly pattern around Ashawayi and Bulong provides an important link between the REA-AIE-derived alteration results and the local deposit geology. Although the Bulong and Ashawayi mineralized systems may differ in fluid source, ore-forming physicochemical conditions, and local mineralization style, both are strongly controlled by fault systems and subsidiary fracture zones. These structures provide favorable pathways for hydrothermal fluid migration and fluid–rock interaction in the shallow crust. Around Ashawayi, hydroxyl-related spectral anomalies are broadly distributed along the nearby fault zone and adjacent lithological contacts, whereas iron-oxide-related spectral anomalies show stronger local concentration. Around Bulong, spectral anomalies are also concentrated near fault intersections and stratigraphic contacts, especially along the northern side of the mineral occurrence. This spatial pattern suggests that both mineralized zones are associated with structurally controlled hydrothermal alteration systems. In such systems, hydroxyl-bearing alteration assemblages, represented by sericitization and chloritization, can develop together with sulfide assemblages dominated by pyrite. Under near-surface weathering and oxidation conditions, Fe-bearing sulfides may be transformed into secondary iron oxide minerals, such as limonite and hematite, thereby producing diagnostic iron oxide-related spectral responses in the visible–near-infrared region of Landsat 8 OLI. Therefore, the coupled distribution of hydroxyl- and iron-oxide-related spectral anomalies around Ashawayi, Bulong, and nearby fault intersections is consistent with the expected surface expression of structurally controlled hydrothermal alteration and supergene oxidation.

The two alteration types also show different geological implications. Hydroxyl-related spectral anomalies are more broadly distributed and form relatively continuous belts or patches along faults and contact zones, especially in the western and central–northern parts of the study area. This broader distribution of hydroxyl-related spectral anomalies may reflect a wider zone of hydrothermal fluid–wall-rock interaction and regional alteration halos. In contrast, iron-oxide-related spectral anomalies are more locally concentrated and commonly appear as clustered or bead-like segments near faults, fault intersections, and mineral occurrence areas. This pattern suggests that the localized iron-oxide-related spectral anomalies may reflect fluid focusing, oxidation, and later near-surface alteration processes. Therefore, hydroxyl- and iron-oxide-related spectral anomalies should be regarded as complementary indicators: the former reflects broader hydrothermal alteration, whereas the latter highlights more localized zones of oxidation and anomaly enrichment.

The southern part of the study area shows a different pattern. Although several faults and stratigraphic units are present, the spectral anomalies are generally weaker and more discontinuous than those in the northern and central–northern areas. This contrast suggests that lithology alone may not fully explain the anomaly distribution. Instead, the spatial association between stronger anomaly concentrations, fault connectivity, and major structural zones is consistent with a structurally influenced hydrothermal alteration pattern.

From an exploration perspective, areas where structural, alteration, and mineralization indicators overlap may represent relatively favorable targets for further field verification. In Figure 16, these areas include the continuous hydroxyl-related spectral anomaly belts along the northern fault system, the locally concentrated iron-oxide-related spectral anomalies near fault intersections, and the anomaly clusters surrounding Ashawayi and Bulong. In particular, zones where hydroxyl- and iron-oxide-related spectral anomalies spatially overlap near major faults, subsidiary fractures, lithological contacts, and known mineral occurrences may be considered potential priority areas for follow-up investigation. Such spatial overlap may indicate structurally favorable sites where hydrothermal fluid migration, wall-rock alteration, sulfide mineralization, and supergene oxidation are spatially associated. This interpretation is locally supported by the petrographic observations in Figure 14 and the site-level assessment results in Section 3.5.3, where hydroxyl-bearing minerals, Fe-oxide assemblages, and sulfide/Cu-polymetallic minerals are shown to be associated with representative samples from the study area. These observations provide additional mineralogical evidence that the REA-AIE-derived anomaly zones are locally associated with hydrothermal alteration and mineralization-related features.

Overall, Figure 16 shows that REA-AIE improves the spatial expression of PCA-derived spectral anomalies and produces results that are broadly consistent with the tectonic and metallogenic framework of the southwestern Tianshan. The spatial distribution of the extracted anomalies may be influenced by regional tectonic evolution, fault activity, lithological contacts, and hydrothermal fluid migration. Therefore, the proposed method may provide useful auxiliary information for identifying potential exploration targets within the selected study area.

Several limitations should be acknowledged. First, training labels were generated from PCA-derived spectral anomalies rather than systematically field-validated mineralogical maps covering the entire study area, which may introduce label noise in spectrally mixed or transitional zones—a limitation shared by most remote sensing-based alteration studies in inaccessible terrains [5,47]. Similarly, the selected background samples should be regarded as unlabeled background rather than confirmed non-alteration samples, because areas without PCA anomaly responses may still contain weak, mixed, or undetected alteration signals. In addition, the selection of anomaly-related PCA components relied on spectral loading characteristics and geological interpretation, which may introduce a degree of analyst dependence. Although consistent selection criteria were applied in this study, automated component-selection approaches and anomaly-threshold sensitivity analyses should be further investigated.

Second, DEM-based topographic correction was not applied in this study. Atmospheric correction and vegetation/snow–ice masking were used to reduce atmospheric and surface-cover interference, respectively, but these procedures do not compensate for slope- and aspect-related illumination differences. Therefore, residual topographic illumination effects may still influence the mapped spectral anomaly responses in this high-relief mountainous area. Future studies should evaluate terrain-corrected reflectance products or topographic normalization approaches to further improve spectral anomaly mapping robustness.

Third, because adjacent 15 × 15 image patches may share overlapping pixels and the validation subset was generated using random stratified sampling, spatial autocorrelation may lead to optimistic sample-level validation metrics. Therefore, the reported Accuracy, F1 Score, Kappa Coefficient, ROC-AUC, and related sample-level metrics should be interpreted as PCA-constrained sample-level comparison metrics rather than spatially independent estimates of spectral anomaly mapping accuracy. In addition, the 15 × 15 input window was selected based on the expected spatial scale of spectral anomaly patterns and the spatial resolution of Landsat 8 OLI, rather than through a systematic multi-scale comparison. Different patch sizes may influence the balance between local spectral–spatial feature extraction and background interference.

Fourth, the comparative and ablation experiments were conducted using a fixed random seed and a single experimental split rather than multiple independent training runs. Therefore, the uncertainty associated with random initialization was not fully quantified. The ablation results also indicate that the contribution of ECA is modest and task-dependent. Thus, ECA should be interpreted as a feature-refinement component that adjusts spectral–spatial feature weighting rather than a factor that uniformly improves all evaluation metrics.

Fifth, the relationship between predicted spectral anomalies, faults, lithological contacts, and known mineral occurrences was mainly interpreted based on spatial correspondence, petrographic evidence, and geological consistency. Quantitative distance-based or enrichment analyses were not conducted in this study. Therefore, the strength of these spatial relationships should be further assessed in future work using quantitative spatial statistics and additional field verification.

Sixth, the public release of some project-derived datasets and implementation materials is restricted, which may limit complete independent reproduction of the present workflow. Nevertheless, the principal PCA pseudo-label generation settings, network architecture, and training configurations are reported in detail in the manuscript to support methodological reproducibility within these constraints.

Finally, the model was developed and evaluated using a single Landsat 8 OLI scene from one study area. Cross-scene, cross-season, cross-sensor, and cross-region generalization were not evaluated in this study. Therefore, the present study should be regarded as a local case application rather than a generally validated operational framework. Future work should test the proposed workflow using multi-temporal images, different sensors, independent geological settings, and more spatially independent validation designs.

## 5. Conclusions

This study developed and evaluated a PCA-guided weakly supervised framework for Landsat 8 OLI-based mapping of hydroxyl- and iron-oxide-related spectral anomalies in the Bulong–Maidan–Tuoyun gold–copper metallogenic belt, South Tianshan. The main conclusions are as follows:

(1) PCA-Guided Weakly Supervised Framework: PCA-derived positive spectral anomaly samples were used as spectral priors to construct pseudo-label samples under limited field-label conditions. This strategy provides a practical weakly supervised approach for spectral anomaly mapping in the selected complex mountainous study area where dense pixel-level field labels are difficult to obtain. By transforming PCA-enhanced anomaly information into structured training samples, the proposed framework establishes a link between conventional spectral enhancement and deep learning-based spectral–spatial refinement.

(2) Residual-ECA Spectral–Spatial Refinement: The REA-AIE model was used to refine PCA-derived anomaly patterns by learning local spectral–spatial features from multispectral image patches. Under the PCA-constrained random sample-level comparison, REA-AIE achieved F1 scores of 95.90% and 97.09% for hydroxyl- and iron-oxide-related spectral anomalies, respectively; these scores quantify agreement with PCA-derived pseudo-labels and are not spatially independent estimates of geological mapping accuracy. The ablation experiment further indicated that the ECA module contributed to channel-wise feature recalibration and affected the precision–recall balance in the two spectral anomaly tasks.

(3) Geological Consistency and Exploration Implications: The refined spectral anomaly maps showed improved spatial continuity and spatial association with fault zones, stratigraphic contact zones, and known mineral occurrences in the Bulong–Maidan–Tuoyun metallogenic belt. Hydroxyl-related spectral anomalies were more widely distributed and may reflect broader hydrothermal alteration halos, whereas iron-oxide-related spectral anomalies were more locally concentrated near structurally favorable zones. Field observations, petrographic evidence, and the petrography-constrained site-level assessment further supported the geological consistency of the predicted anomaly zones. For the union assessment, REA-AIE anomalies occurred within 90 m of 43 of the 53 petrographically verified altered sites, while no anomaly was recorded within 90 m of the 30 non-altered control sites under the adopted assessment criterion. Because the 83 sites represent a selected and spatially limited subset, this assessment should be interpreted as evidence of geological consistency at the sampled locations rather than as complete validation of the full-area anomaly maps. These results support the use of the mapped anomaly zones as auxiliary information for follow-up field verification in the selected study area. However, the spatial relationships among anomalies, faults, lithological contacts, and mineral occurrences should be further evaluated using quantitative spatial statistics.

Because the framework was evaluated using a single Landsat 8 OLI scene from one geological setting, its transferability should be further tested across additional scenes, seasons, sensors, and regions.

## Figures and Tables

**Table 1:** Complete PCA loading matrix and explained variance ratios for hydroxyl-related spectral anomaly extraction.

PC	Explained Variance (%)	Band 2	Band 5	Band 6	Band 7
PC1	98.3521	0.2418	0.5214	0.6129	0.5423
PC2	1.1900	0.8284	0.3260	−0.3741	−0.2600
PC3	0.3581	0.4671	−0.7574	0.0600	0.4522
**PC4**	**0.0999**	**−0.1928**	**0.2195**	**−0.6934**	**0.6587**

Note: Bold values indicate the selected principal component (PC4) and its corresponding loading values used for hydroxyl-related spectral anomaly extraction.

**Table 2:** Complete PCA loading matrix and explained variance ratios for iron-oxide-related spectral anomaly extraction.

PC	Explained Variance (%)	Band 2	Band 4	Band 5	Band 6
PC1	98.0125	0.2583	0.4685	0.5503	0.6411
PC2	1.5686	0.6480	0.4409	0.0423	−0.6196
PC3	0.3159	0.5997	−0.1940	−0.6356	0.4457
**PC4**	**0.1029**	**−0.3921**	**0.7406**	**−0.5398**	**0.0801**

Note: Bold values indicate the selected principal component (PC4) and its corresponding loading values used for iron-oxide-related spectral anomaly extraction.

**Table 3:** Sample-level agreement with PCA-derived pseudo-labels for hydroxyl- and iron-oxide-related spectral anomaly identification.

Task	Model	Accuracy (%)	Precision (%)	Recall (%)	F1 Score (%)	Kappa Coefficient (%)
Hydroxyl-related spectral anomaly	REA-AIE	95.88	95.48	96.32	95.90	91.76
	ResNet	94.89	93.90	96.01	94.95	89.78
	U-Net	91.83	93.70	89.70	91.65	83.67
	CNN	94.54	92.71	96.69	94.66	89.09
Iron-oxide-related spectral anomaly	REA-AIE	97.05	95.74	98.48	97.09	94.10
	ResNet	96.08	94.96	97.33	96.13	92.17
	U-Net	95.97	95.42	96.58	96.00	91.94
	CNN	96.17	94.66	97.86	96.23	92.34

**Table 4:** PCA-constrained sample-level comparison results of the ablation experiment for the hydroxyl- and iron-oxide-related spectral anomaly tasks.

Task	Variant	Accuracy (%)	Precision (%)	Recall (%)	F1 Score (%)	Kappa Coefficient (%)
Hydroxyl-related spectral anomaly	Res-only	95.79	95.73	95.86	95.79	91.58
	Res + ECA	95.88	95.48	96.32	95.90	91.76
	Res + SE	95.32	94.33	96.25	95.28	90.64
Iron-oxide-related spectral anomaly	Res-only	96.40	97.26	95.48	96.36	92.79
	Res + ECA	97.05	95.74	98.48	97.09	94.10
	Res + SE	96.49	96.70	96.27	96.48	92.98

**Table 5:** PR-AUC values and threshold-dependent mapped-area statistics for the REA-AIE prediction-score maps.

Task	PR-AUC	Prediction-Score Threshold	Spectral Anomaly Pixels	Spectral Anomaly Area (km^2^)	Spectral Anomaly Area Percentage (%)
Hydroxyl-related spectral anomaly	0.4582	0.5	318,597	286.7373	5.6573
		0.6	292,558	263.3022	5.1950
		0.7	265,319	238.7871	4.7113
		0.8	234,092	210.6828	4.1568
		0.9	190,188	171.1692	3.3772
Iron-oxide-related spectral anomaly	0.5457	0.5	271,319	244.1871	4.8178
		0.6	247,254	222.5286	4.3905
		0.7	222,810	200.5290	3.9564
		0.8	195,119	175.6071	3.4647
		0.9	158,107	142.2963	2.8075

Note: Thresholds refer to uncalibrated prediction scores. PR-AUC was calculated from the continuous prediction scores against the full-area PCA-derived reference labels. Mapped area was calculated using the 30 m × 30 m pixel size of Landsat 8 OLI, and the final maps used a prediction-score threshold of 0.7.

**Table 6:** Petrography-constrained site-level assessment of hydroxyl-bearing alteration sites, iron oxide alteration sites, and their union using a 90 m buffer.

Method	Assessment	TP	FN	TN	FP	Recall (%)	Balanced Accuracy (%)	Recall 95% CI (%)
PCA	Hydroxyl	30	12	41	0	71.43	85.71	56.43–82.83
	Iron oxide	28	11	44	0	71.79	85.90	56.22–83.46
	Union	38	15	30	0	71.70	85.85	58.43–82.03
REA-AIE	Hydroxyl	34	8	41	0	80.95	90.48	66.70–90.02
	Iron oxide	32	7	44	0	82.05	91.03	67.33–91.02
	Union	43	10	30	0	81.13	90.57	68.64–89.41

Note: PCA denotes the median-filtered PCA-derived anomaly baseline. No false-positive detections were observed among the selected site-level negative samples under the adopted 90 m buffer criterion; therefore, Precision and Specificity were 100% for all methods and assessment tasks. Recall 95% confidence intervals were calculated using the Wilson method.

## Data Availability

The data presented in this study are available on request from the corresponding author due to project-related data management restrictions.
